# Concordant Signal of Genetic Variation Across Marker Densities in the Desert Annual *Chylismia brevipes* Is Linked With Timing of Winter Precipitation

**DOI:** 10.1111/eva.70046

**Published:** 2024-12-16

**Authors:** Daniel F. Shryock, Nila Lê, Lesley A. DeFalco, Todd C. Esque

**Affiliations:** ^1^ U.S. Geological Survey Western Ecological Research Center Boulder City Nevada USA; ^2^ California Botanic Garden Claremont California USA

**Keywords:** adaptation, climate change, genome scan, isolation by environment, restoration, seed sourcing

## Abstract

Climate change coupled with large‐scale surface disturbances necessitate active restoration strategies to promote resilient and genetically diverse native plant communities. However, scarcity of native plant materials hinders restoration efforts, leading practitioners to choose from potentially viable but nonlocal seed sources. Genome scans for genetic variation linked with selective environmental gradients have become a useful tool in such efforts, allowing rapid delineation of seed transfer zones along with predictions of genomic vulnerability to climate change. When properly applied, genome scans can reduce the risk of maladaptation due to mismatches between seed source and planting site. However, results are rarely replicated among complimentary data sources. Here, we compared RAD‐seq datasets with 819 and 2699 SNPs (in 625 and 356 individuals, respectively) from the Mojave Desert winter annual *Chylismia brevipes* . Overall, we found that the datasets consistently characterized both neutral population structure and genetic–environmental associations. Ancestry analyses indicated consistent spatial genetic structuring into four regional populations. We also detected a marked signal of isolation by resistance (IBR), wherein spatial genetic structure was better explained by habitat resistance than by geographic distance. Potentially adaptive loci identified from genome scans were associated with the same environmental gradients—fall precipitation, winter minimum temperature, and precipitation timing—regardless of dataset. Paired with our finding that habitat resistance best explained genetic divergence, our results suggest that isolation of populations within environmentally similar habitats—and subsequent local adaption along gradients parallel to these habitats—drive genome‐wide divergence in this species. Moreover, strong genetic associations with winter precipitation timing, along with forecasted shifts in precipitation regime due to midcentury climate change, could impact future population dynamics, habitat distribution, and genetic connectivity for *C. brevipes* populations within the Mojave Desert.

## 1 Introduction

Ecological restoration of native plant communities is increasingly needed to counter large‐scale surface disturbances, invasive species, altered fire regimes, and impacts from climate change. In the western United States, the use of restoration treatments with native species has risen in recent decades (Copeland et al. 2018), along with programmatic efforts to create a sustainable supply of native seeds (Haidet and Olwell 2015; Oldfield and Olwell 2015). However, restoration treatments in the typically arid environments of this region have frequently failed (Knutson et al. 2014), in part due to extreme environmental filters including seed predation (Defalco et al. 2012), invasive species (Abella et al. 2012; DeFalco et al. 2003), and unpredictable precipitation (Chesson et al. 2004). Strong evidence suggests that local adaptation plays a key role in population persistence in this region (Baughman et al. 2019), in some cases leading to rapid shifts in traits under selection (Goergen, Leger, and Espeland 2011; Leger and Goergen 2017). Genotypes maladapted to a restoration site may fail to establish or cause outbreeding depression in the local population (Hufford and Mazer 2003; McKay et al. 2005). Even when seed transfer distances are minimal, rapid climate change poses an additional challenge that may disrupt gene–environment associations (GEA), reducing local population fitness and potentially favoring alternative alleles (Capblancq et al. 2020). Knowledge of GEA and gradients in local adaptation, particularly in relation to gene flow, population history, and climate (Massatti et al. 2018), is fundamental to guiding seed‐sourcing decisions. However, acquiring landscape‐scale genetic information from priority restoration species is a persistent challenge for native plant materials development programs.

Genome scans for natural selection are a useful tool in native plant restoration, allowing practitioners to identify potential locally adapted genotypes from cost‐efficient, reduced representation sequencing datasets in non‐model species (e.g., Shryock et al. 2021). These techniques seek to identify loci influenced by selection either as divergence outliers (e.g., Foll and Gaggiotti 2008) or through environmental association analysis (GEA; see Rellstab et al. 2015 for a review), and while they may not identify the true targets of selection (de Villemereuil et al. 2014; Yoder and Tiffin 2018), it is assumed that the loci identified through genome scans are linked with adaptive genetic variation. For this reason, these loci are typically referred to as “potentially adaptive” until verified through additional means, such as common garden or reciprocal transplant experiments. In environments where such experiments are challenging, such as in deserts where drought years frequently occur, genome scans can facilitate development of restoration seed sourcing guidelines for a broader range of species. For example, environment‐associated genetic variation may be used to create seed transfer zones, which aim to identify geographic areas within which seeds may be transferred with limited risk of maladaptation and which are a frequent target of management efforts in the western United States (Johnson et al. 2010).

It should be noted that genome scans on reduced representation sequencing data have received scrutiny, given that they directly sample a small portion of the genome (Lowry et al. 2017). However, genomic representation increases markedly with even moderate linkage disequilibrium (McKinney et al. 2017) and can increase due to other processes such as divergence hitchhiking (Yoder and Tiffin 2018). Similarly, isolation by environment (IBE) is pervasive (Shafer and Wolf 2013) and affects genome‐wide variation such that neutral population structure may mirror adaptive variation (Nosil, Funk, and Ortiz‐Barrientos 2009), particularly in regions with extreme topography and climatic gradients that affect dispersal (e.g., Massatti and Knowles 2020). Techniques for reducing false positives in genome scans have also considerably advanced, with simulation studies generally indicating acceptable rates of Type I error (De Mita et al. 2013; de Villemereuil et al. 2014; Vilas, Pérez‐Figueroa, and Caballero 2012), particularly for multivariate techniques (Forester et al. 2016, 2018). Of greatest consequence for restoration practitioners, studies have frequently found convergent results between genome scans and common garden experiments (De Kort et al. 2014; Fournier‐Level et al. 2011; Hancock et al. 2011; Herrera, Medrano, and Bazaga 2015; Richardson, Rehfeldt, and Kim 2009; Steane et al. 2014) and genome scans on RAD‐seq data can be used to identify candidate genes under selection when mapped to a reference (e.g., Larson et al. 2017).

An issue for which there is more limited guidance concerns the quality and density of reduced representation sequencing data needed to effectively guide restoration. RAD‐seq data have known biases in allele frequency estimation when polymorphisms occur at restriction sites (Arnold et al. 2013), and population genetic inferences can vary due to the choice of bioinformatic pipeline (Shafer et al. 2017) or SNP filtering parameters (e.g., minor allele frequency thresholds) (O'Leary et al. 2018; Linck and Battey 2019). Genome scans in a restoration context are often used to develop ecological inferences regarding the relative importance of environmental gradients (precipitation and temperature) in driving local adaptation among populations or in shaping patterns of landscape resistance and gene flow. Previous evidence suggests that bioinformatic filtering parameters affect the identification of loci under selection (Ahrens et al. 2021), but the degree to which such inferences are robust to differences in genetic datasets warrants further scrutiny, particularly for reduced representation sequencing data on species lacking a reference genome.

Here, we compare complementary RAD‐seq datasets in a nonmodel species of restoration importance, the desert annual *Chylismia brevipes* , which was recently listed as a priority species for seed collection and increase efforts in the Mojave Desert (Esque et al. 2021). *C. brevipes* is pollinated by native oligolectic bees (e.g., *Andrena*) (Esque et al. 2021; Raven 1979) and features in the diet of the Mojave desert tortoise ( *Gopherus agassizii* ; Esque 1994), a species listed as threatened under the U.S. Endangered Species Act (55 FR 12178). Development of seed transfer zones for *C. brevipes* would better facilitate its use in desert restoration. Here, we incorporate multiple analyses, including GEAs and ancestry analyses, to derive seed transfer zones for management of *C. brevipes* in the Mojave Desert. Given that variation in germination and phenology of winter annuals is largely tied to the timing of fall and winter rains (Beatley 1974), we hypothesized that the seasonality of rainfall in the fall–winter–spring seasons would likewise drive within‐species adaptive divergence. We also examine the genomic offset of study populations with respect to predicted changes in climate (Capblancq et al. 2020; Rellstab, Dauphin, and Exposito‐Alonso 2021), revealing regions most at risk of disruption of genotype—environment associations. Throughout our analyses, we compare results across independently sequenced RAD‐seq datasets, focusing largely on issues that could impact management, such as the strength and direction of SNP—environment associations, and how sample locations are assigned to genetic populations.

## 2 Methods

### 2.1 Study Site and Species

Our study was conducted in the Mojave Desert ecoregion with populations sampled from California, Nevada, and Arizona. This warm‐desert ecoregion spans approximately 130,000 km^2^ and is characterized by extreme physiographic and climatological gradients. A series of north‐to‐south trending mountain ranges create broad gradients in precipitation and temperature, with interlaying basins marked by largely dry washes (except following heavy rainfall), alluvial fans, and playas. Annual precipitation follows elevation gradients, ranging from 50 to 300 mm and averaging 137 mm. In the western Mojave Desert, precipitation occurs largely in the winter/early spring months (November—March). However, the eastern Mojave Desert experiences a more bimodal precipitation regime, with greater summer/fall precipitation due to tropical storm tracks from the Gulf of Mexico (Hereford, Webb, and Longpré 2006). Mean annual temperature (MAT) is approximately 17°C but ranges from < 0°C in winter to over 50°C in summer, with highest temperatures at lower elevations.

*C. brevipes* (A. Gray), or yellow cups, is a short‐statured, rosette‐forming winter annual in the family *Onagraceae*. Study samples were predominantly identified as *C. brevipes* ssp. *brevipes* , but subspecies identifications were not always definitive, and *C. brevipes* ssp. *brevipes* is known to intergrade with less common varieties including ssp. *pallidula* and ssp. *arizonica*. The species germinates in response to late‐fall or winter rainfall and typically flowers from March through May. *C. brevipes* is an obligate outcrosser. No mechanism for long‐distance seed dispersal appears to be present, but passive dispersal from dehiscing capsules is likely. Habitat occurs primarily in creosote bush ( *Larrea tridentata* ) scrub or Joshua tree ( *Yucca brevifolia* and *Y. jaegeriana* ) woodland on sandy desert slopes and dry washes throughout the Mojave Desert and south into the northern sections of Sonoran Desert and is typically from 300 to 1000 m in elevation. While this study did not assess ploidy, *C. brevipes* is presumed to be diploid (2n = 14; Raven 1962, 1969). No genome size estimate is available, but species in closely related genera show an average size of only 0.94 pg. (for genus *Oenothera*; https://cvalues.science.kew.org/).

### 2.2 Environmental Variables

We derived a suite of environmental variables that could potentially drive adaptive genetic variation among populations of *C. brevipes* (Table 1; Appendix S1). Climate variables were derived using ClimateNA v. 7.3 (Wang et al. 2016), which downscales PRISM data (Daly et al. 2008) using local elevation adjustments. We included precipitation variables reflecting fall rainfall (September—October), total winter rainfall (November–April), and the ratio of rainfall occurring in late winter (*WPratio*). Snowfall is infrequent at low and middle elevations in the Mojave Desert and was not included. Annual precipitation seasonality (PCV) was strongly correlated with the seasonality of rainfall in the winter months alone; therefore, we included only the former variable in analyses. NDVI amplitude, a measure of annual green‐up potential above the baseline NDVI (normalized difference vegetation index), was derived from the USGS eMODIS Remote Sensing Phenology network (https://doi.org//10.5066/F7PC30G1). Topographic metrics were calculated using a 30 m^2^ digital elevation model from the USGS National Elevation Dataset (http://ned.usgs.gov) and subsequently aggregated to a 1 km^2^ resolution. We did not consider elevation in our analysis because this variable was strongly correlated with a number of other climate variables, including MAT and summer maximum temperature (SMT). Soil variables were chosen to reflect soil surface texture and bulk density, which moderate plant available water and influence traits such as water use efficiency (Ehleringer and Cooper 1988; Smith et al. 1995). These variables were downloaded and mosaicked from the SoilGrids 2.0 database (Poggio et al. 2021). All variables were analyzed at a 1 km^2^ spatial resolution.

**TABLE 1:** Environmental variables included in landscape genetic models.

Environmental variable	Code	Definition
Climate		
Fall precipitation (mm)	Fall.PPT	Average precipitation received from Sept to Oct
Winter precipitation (mm)	WP	Average precipitation received from Nov to April
Precipitation seasonality (%)	PCV	Coefficient of variation in monthly precipitation totals
Winter precipitation ratio (%)	WPratio	Ratio of late (Feb–Apr)/total (Nov–April) winter precipitation
Summer maximum temperature (°C)	SMT	Maximum temperature of warmest month
Winter minimum temperature (°C)	WMT	Minimum temperature of coldest month
Diurnal temperature range (°C)	DTrange	Mean of the monthly temperature ranges (monthly maximum minus monthly minimum)
*Satellite metrics*		
NDVI amplitude	AMP	Maximum increase in canopy photosynthetic activity above the baseline averaged for the period 2003–2017
Bulk density (cg/cm^3^)	BD	Bulk density of the fine earth fraction
Sand (g/kg)	Sand	Proportion of sand particles (> 0.05 mm) in the fine earth fraction (0–5 cm depth)
Topography		
Heat load index	HLI	Aspect/slope transformation index (McCune and Keon 2002) representing the range in heat load from coolest (northeast slope) to warmest (southwest slope).
Topographic position index	TPI	Steady‐state wetness index expressed as a function of slope and upstream contributing area

*Note:* Variables were derived at 1 km^2^ resolution. Climate variables are averages for the reference period 1980–2010.

### 2.3 DNA Extraction and RAD‐seq Genotyping

Green leaf tissues were collected, dried using silica, and sent to the California Botanic Garden (formerly Rancho Santa Ana Botanic Garden) in Claremont, CA, for processing. We sampled approximately 12–15 plants per location, spaced at least 10 m apart, for a total of 760 individuals from 66 locations throughout the Mojave Desert (Appendix S1). We used approximately 10 mg of field‐collected, silica‐dried leaf tissue samples and a modified CTAB protocol (Doyle and Doyle 1987), where the main modification included the addition of a pectinase step, to extract DNA. Gel electrophoresis and Qubit 2.0 Fluorometer (Invitrogen) assays indicated high quality and quantities of all DNA samples. Extracted DNA was fluorometrically quantified and diluted to a standard concentration of 20 ng/μL for genomic library preparation.

Restriction site‐associated DNA‐sequencing (RAD‐seq) libraries were prepared and sequenced by Floragenex (9590 Southwest Gemini Drive, Beaverton, OR, 97008). Due to the inherent difficulties of obtaining annual plant tissue collections across the Mojave Desert ecoregion during prolonged drought, the first set of samples was sequenced in December 2018 and the latter set of samples was sequenced in March 2020. These collections included a total of 760 samples prepared with a *SbfI* enzyme digest; 380 of these samples were also prepared separately with a *PstI* enzyme digest in 2020. Floragenex performed multiple steps: a spot QA/QC of genomic DNA; ligation of RAD adapters to associate sequence reads to individuals; sonicated, performed end repair, and ligated Illumina sequencer adapters; size‐selected targeting within a 300–500 bp window; amplified using polymerase chain reaction (PCR) and purified those PCR reactions; and combined the final library. They completed a Bioanalyzer (Agilent) trace to validate library integrity, and a quantitative PCR (qPCR) was run to estimate optimal NGS plating concentration prior to sequencing. For both datasets, single‐end sequencing was run across eight fully allocated lanes of a 1 × 100 bp Illumina HiSeq4000 platform (380 samples per run), with the samples run in replicate to improve depth of coverage.

### 2.4 Bioinformatics and Quality Filtering

We used the ipyrad 0.9.17 to demultiplex sequence reads (Eaton and Overcast 2020) and the STACKS 2.5 (Catchen et al. 2013) pipeline to build a *de novo* assembly. We conducted parameter optimization for the *de novo* assembly by running ustacks with a subset of four individuals across all populations, totaling 275 representative individuals, using the r80 method that varies parameters to maximize the number of polymorphic loci shared in 80% of the individuals (Paris, Stevens, and Catchen 2017). We explored *M*, the number of mismatches allowed between stacks (i.e., putative alleles) before merging into a putative locus, at a range from *M* = 3 to 8; *n*, the number of mismatches allowed during the construction of the catalog, and was held equivalent to *M* for each optimization test. The minimum number of raw reads required to form a stack (*m*) was consistently held at three (Rochette and Catchen 2017). *De novo* assembly was then conducted for the entire dataset using ustacks and the catalog of loci was compiled with the subset of 275 representative individuals using cstacks. The optimization *M* = *n* = 4, *m* = 3 was retained for downstream analyses for both datasets. The Stacks *Populations* module was used to generate final genotypes with additional filters of r = 0.5 for the minimum percentage of individuals in a population required to process a given locus for that population and max_observed_het = 0.7 (maximum observed heterozygosity). Genotype calls from *Populations* were exported to a VCF (variant call format) for additional filtering.

Using the VCF exported from Stacks, we applied the following additional filters using the R package “vcfR” (Knaus and Grünwald 2017): no more than 30% missing data for individuals, and a minor allele frequency count of at least three. Additionally, we filtered SNPs with excessive depth above the 99th quantile (approximately 80× for the *PstI* data, and 450× for the *SbfI* data). Finally, because many analyses assume unlinked loci, we filtered the VCFs to one random SNP per RAD locus.

### 2.5 Genetic Population Structure

Summaries of genetic diversity within sample locations were computed using the Stacks *Populations* module (Appendix S2). Additionally, we compared within‐ and among‐population variation for each dataset with an analysis of molecular variance (AMOVA) using the R package “poppr” (Kamvar et al. 2014). We compared spatial population structure across both datasets using the nonparametric discriminant analysis of principal components (DAPC), implemented in the R package “adegenet” (Jombart, Devillard, and Balloux 2010). Prior group assignments for DAPC were determined using “find.clusters,” which uses k‐means clustering of PC‐transformed genotypes. The optimal grouping was selected via the Bayesian information criterion (BIC). Additionally, we selected an optimal number of PCs to retain in the DAPC using cross‐validation withholding 20% of populations (adegenet function “xvalDAPC”). For the larger *SbfI* dataset, we also computed individual ancestry coefficients with the Bayesian program STRUCTURE (Pritchard, Stephens, and Donnelly 2000) using the correlated allele frequencies model with K ranging from 1 to 7. Each STRUCTURE run consisted of a burn‐in of 150,000 and run length of 250,000. We used the Delta K method to identify the best‐supported number of clusters (Evanno, Regnaut, and Goudet 2005) and aligned and visualized results using “Clumpak” and “Distruct,” respectively (Kopelman et al. 2015; Rosenberg 2004). We further visualized spatial genetic structure in the *SbfI* dataset by creating a spatial interpolation of pairwise population *F*_ST_ values using the R package “MAPI” v. 1.0.1 (Piry et al. 2016). *F*_ST_ was calculated following Weir and Cockerham 1984, with the R package “hierfstat” (Goudet and Jombart 2022). Finally, we created a map of ancestral probability surfaces for the *SbfI* data using the R package “POPMAPS” (Massatti and Winkler 2022) with the following parameters: num_tested = 3 and *popmod* = −0.001 (see Massatti and Winkler 2022 for details). *POPMAPS* assigns grid cells to their most likely population of origin by combining previous genetic population assignments with an inverse distance–weighted (IDW) genetic distance calculated on a user‐supplied pairwise resistance matrix. For pairwise resistance values, we used least cost distances calculated from a habitat transition layer created with the R package “gdistance” (van Etten 2017). Habitat probabilities were based on a previously developed species distribution model (SDM) for *C. brevipes* in the Mojave Desert (Shryock, DeFalco, and Esque 2022; https://doi.org/10.5066/P9XQJFEL).

### 2.6 Genome Scans for Selection

We used a combination of genome scan algorithms to detect loci potentially linked with selection along environmental gradients (hereafter referred to as “potentially adaptive loci”), including one *F*_ST_ outlier detection approach and one GEA approach. To identify *F*_*ST*_ outliers (i.e., those with an *F*_ST_ larger than expected under neutrality, and potentially under selection), we used the Bayesian approach BayeScEnv version 1.1 (de Villemereuil and Gaggiotti 2015). This program updates the widely used and robust F‐model of BayeScan (Foll and Gaggiotti 2008) to incorporate a locus‐specific effect of selection along an environmental gradient, resulting in a lower false‐positive rate than the original algorithm in multiple demographic scenarios (de Villemereuil and Gaggiotti 2015). We ran BayeScEnv for each potential environmental variable (Table 1) and each dataset (*PstI* and *SbfI* markers) using program defaults and sample locations as populations. Adjusted q‐values were used to identify significant outlier loci.

For the GEA approach, we used a multivariate redundancy analysis (RDA) to identify loci showing larger‐than‐expected associations with environmental gradients. By regressing SNPs with environmental variables in multivariate ordination space, RDA can effectively distinguish loci with stronger environmental associations than are present in the background genomic variation (e.g., population structure). In simulations, the technique has shown a low false‐positive rate coupled with high power to detect loci under selection (Forester et al. 2016, 2018). Following Forester et al. (2016), we used a conservative threshold to select potentially adaptive loci, including those with RDA axis scores more than 3 standard deviations from the mean. We fit RDA to the individual allele frequencies using the R package “vegan” version 2.4–6 (Oksanen et al. 2018). Only significant RDA axes were considered in the detection of potentially adaptive loci based on the permutation test implemented in the “vegan” package (function “anova.rda”). Additionally, we included only environmental variables with variance inflation factors < 10 (“vif.cca” function) in the model to avoid issues of multicollinearity, which can affect RDA.

### 2.7 Resistance Surface Modeling

Following a previously proposed framework (Orsini et al. 2013; Shryock et al. 2021), we applied causal modeling (Cushman et al. 2006) to relate genetic divergence with several underlying processes potentially affecting gene flow: isolation by distance (IBD; divergence increases among geographically separated populations due to neutral genetic drift; Wright 1943); isolation by habitat resistance (IBR; genetic divergence increases with resistance distance between habitat patches due to limitations of dispersal into unsuitable habitat; McRae 2006); and IBE (environmental selection shifts allele frequencies, increasing divergence at selected loci and linked sites due to reduced gene flow between environmentally dissimilar populations; Orsini et al. 2013). Importantly, IBE can become genome‐wide if selection creates strong barriers to gene flow, increasing divergence hitchhiking as well as drift at neutral loci; this latter phenomenon has been alternatively termed isolation by ecology (Shafer and Wolf 2013) or isolation by adaptation (Nosil, Funk, and Ortiz‐Barrientos 2009; Orsini et al. 2013). Given the larger sample size and more even distribution of populations across our study region, we performed these analyses exclusively on the *SbfI* dataset. We use Mantel and partial Mantel tests, conducted separately on neutral and potentially adaptive loci, to test each hypothesis. For example, under IBR, we would expect partial Mantel tests showing positive associations to habitat resistance in both neutral and potentially adaptive loci after removing the effects of geographic and environmental distance. In all tests, genetic distances were calculated as population pairwise *F*_ST_ values (Weir and Cockerham 1984) using the R package “hierfstat” (Goudet 2005). Geographic distance was calculated as a pairwise Euclidean distance matrix between UTM‐projected population coordinates. Environmental distance was calculated as a pairwise Euclidean distance matrix calculated on standardized environmental variables (Table 1). To derive habitat resistances, we used the SDM for *C. brevipes* (described above) as input to the R package “gdistance.” Using this package, we transformed the SDM into a “TransitionLayer” and calculated pairwise cost distances between populations. In preliminary analyses, we compared both least‐cost resistance distances and commute distances, the latter of which is equivalent to the circuit‐based distance of McRae (2006). Least cost distances better explained genetic distances between populations than commute distances and were retained for all subsequent analyses. In order to avoid bias (Legendre and Fortin 2010), we evaluated Mantel tests based on the strength of the correlation statistic, r, rather than the permutational p‐values (Cushman et al. 2013; Shirk, Landguth, and Cushman 2018).

### 2.8 Multivariate Models of Potentially Adaptive Loci

We compared two multivariate, nonlinear approaches for mapping allele frequency turnover across the landscape and identifying important environmental gradients. First, we fit gradient forest (GF) models to both the *SbfI* and *PstI* datasets of potentially adaptive loci, using minor allele frequency matrices as the response variable (Fitzpatrick and Keller 2015; Gougherty, Keller, and Fitzpatrick 2021). A multivariate extension of random forests, GF fits allele frequencies as nonlinear functions of environmental gradients by creating an ensemble of random forest regression trees for each SNP, retaining those with predictive value, and subsequently calculating a cumulative, monotonic turnover function for each predictor (Ellis, Smith, and Roland Pitcher 2012). Rapid increases in the turnover functions occur where the split importance values from the ensemble of regression trees explain the most genetic variation on either side of the “split” in environmental predictor values. Variable importance can be measured as the decrease in performance when each predictor is randomly permuted. We used GF to compare the relative importance and turnover functions of environmental predictors between our two datasets. We fit GF using the R package “gradientForest” with 2000 random trees and a correlation threshold of 0.7 for variable partitions (Ellis, Smith, and Roland Pitcher 2012).

Second, we fit generalized dissimilarity models (GDM) to the allele frequencies for potentially adaptive loci from each dataset using the R package “gdm” (Manion et al. 2018). GDM, a multivariate extension of permutational matrix regression, models pairwise dissimilarities (genetic distance) between sites as nonlinear functions of environmental predictors (see Ferrier et al. 2007, for details; Fitzpatrick and Keller 2015, for application to genomics). In this approach, curvilinear relationships between genetic distances and environmental dissimilarities are modeled by fitting monotonic I‐spline functions to each predictor reflecting the rate and magnitude of turnover in genetic distance across the predictor gradient. A strength of GDM is that it allows comparisons of distance‐based hypotheses regarding spatial patterns of genetic divergence, similar to our causal modeling approach. Hence, we compared GDM models with environmental predictors alone, with geographic distance, and with habitat resistance (resistance distance matrix described above). As GF and GDM results were similar between the *SbfI* and *PstI* datasets, we evaluated alternative GDMs incorporating resistance distances only on the *SbfI* dataset, which afforded much greater sample size given the number of populations (*n* = 62 vs. *n* = 35 for *PstI*). However, GDMs incorporating environmental predictors were evaluated on both datasets.

### 2.9 Seed Transfer Zones

We used habitat resistance values in combination with the best‐fit GDM of potentially adaptive allele frequencies to delineate seed transfer zones for *C. brevipes* within the Mojave Desert based on the larger *SbfI* dataset. First, we used the GDM to predict pairwise population genetic dissimilarities in the form of a distance matrix, on which we then performed a hierarchical agglomerative cluster analysis with Ward's linkage method to group sample locations into genetic clusters with similar potentially adaptive allele frequencies. We selected cut points to trim the dendrogram with four and six genetic clusters based on both the silhouette statistic (Rousseeuw 1987) and permutational MANOVA (function “adonis” in the R package “vegan”; Oksanen et al. 2018). Next, to extrapolate the genetic clusters across the full Mojave Desert ecoregion, we used a nearest‐neighbor algorithm combining habitat resistance and genetic distance. In the algorithm, multivariate cost distances from each raster cell to each genetic sample location were calculated as a combination of (1) an accumulated cost distance based on least‐cost habitat resistances, calculated with the R “gdistance” package on the SDM for *C. brevipes* ; and (2) a multivariate Euclidean distance from the origin point calculated on GDM‐transformed environmental predictors, using the “gdm.transform” function in the R “gdm” package. The cost measures were weighted according to their relative importance in the GDM model, as follows: 0.6 × (Habitat resistance) + 0.4 × (Environmental distance). Finally, each raster cell was assigned to the same genetic cluster as the sample location to which it had the minimum overall cost distance. This resulted in either four or six seed transfer zones, based on the dendrogram cut points of four and six genetic clusters. To avoid extrapolation into unsuitable habitats while still accounting for uncertainty in the SDM due to incomplete locality data, we clipped seed transfer zones to areas with a habitat probability of at least 0.2, thereby including marginal habitat where *C. brevipes* may be present but has not been recorded in public databases.

### 2.10 Future Projection

Genomic offset is a measure of the disruption in GEAs due to climate change, which could lead to maladaptation if populations do not rapidly migrate or adapt (Capblancq et al. 2020). For GDM, genomic offset is defined as the difference in predicted genetic distances for current versus future transformed predictors at a location, reflecting the degree to which a local population would have to shift allele frequencies in order to track changing climate. We used the “predict.gdm” function in the R “gdm” package to calculate genomic offset for the present climate (1980–2010 normal) versus the 2041–2070 normal period. Climate variables for the future climate scenario were derived in ClimateNA v 7.3 (Wang et al. 2016) using an 8‐algorithm general purpose global circulation model (GCM) ensemble (Mahony et al. 2022) and the IPCC moderate‐high emission scenario (CMIP6, SSP3‐7.0; Arias et al. 2021). The eight‐model GCM ensemble was selected by Mahony et al. (2022) such that projections would remain consistent with the IPCC's “very likely” range of equilibrium climate sensitivity.

We also sought to project seed transfer zones into future climate space, displaying for each zone the area of similar future climate still bounded by the Mojave Desert ecoregion. For this calculation, we used the nearest‐neighbor algorithm described above with modified inputs. First, we reprojected the *C. brevipes* SDM into future climate space by incorporating future climate predictors into the model projections, leaving nonclimate predictors constant. Similarly, we created GDM‐transformed future climate predictors using the best‐fit GDM. With these future‐projected inputs, we repeated the nearest‐neighbor algorithm to assign grid cells to their closest population, shifting zonal boundaries according to differences in predicted habitat connectivity and climate. Future‐projected seed zones were again clipped to areas with a future habitat probability ≥ 0.2 based on the future‐projected SDM.

## 3 Results

### 3.1 Sequencing and Quality Filtering

**FIGURE 1:**
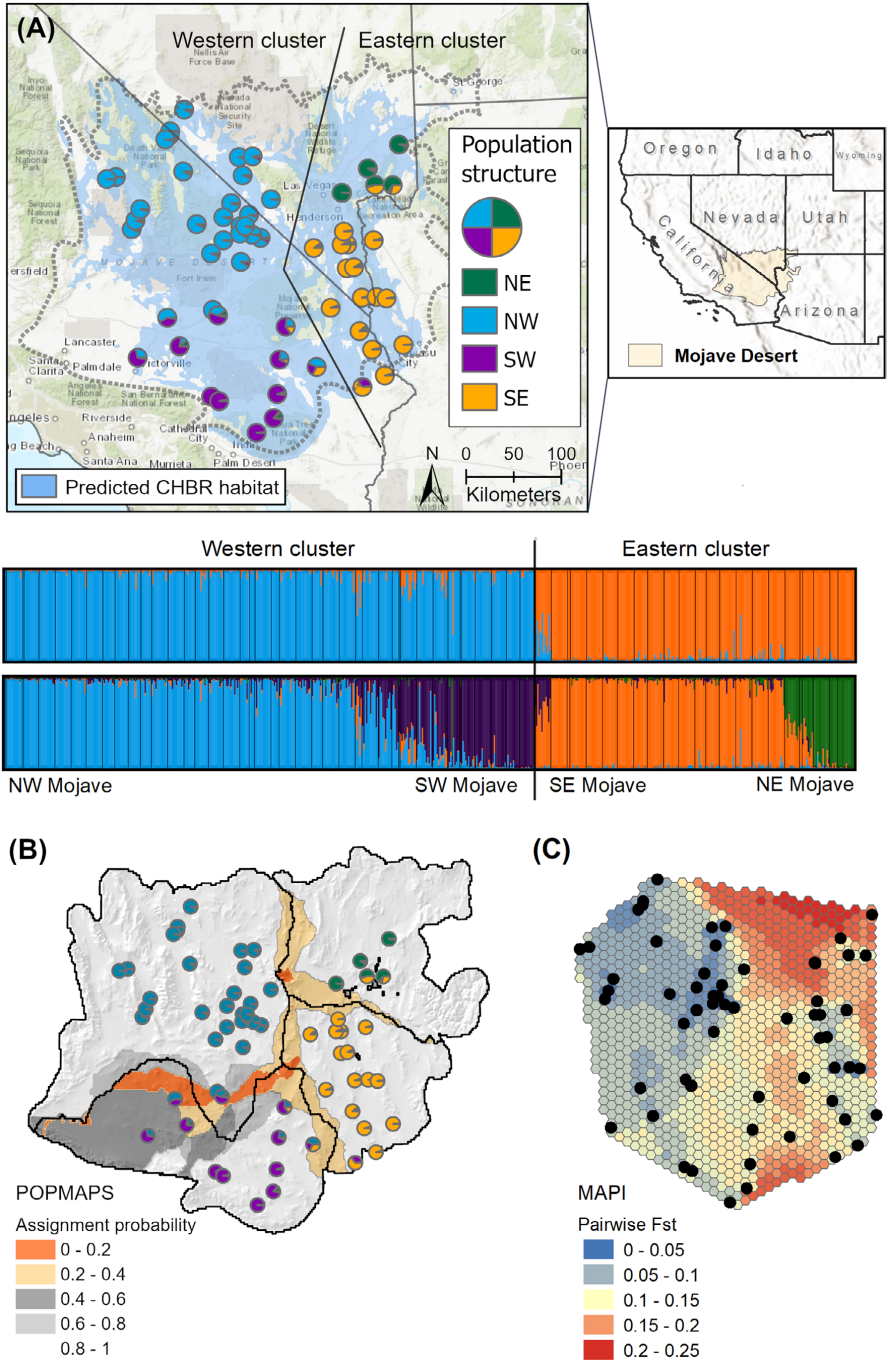
Sampling locations and genetic population structure for *Chylismia brevipes* in the Mojave Desert (values shown are from the *SbfI* dataset). (A) We assessed a total of 62 sampling locations for genetic ancestry, resulting in four geographic populations separated along a central longitudinal cline based on results from *STRUCTURE*. (B) A *POPMAPS* analysis indicated transitional zones of likely genetic admixture (i.e., low probability of population assignment) between the genetic populations. (C) Interpolations of pairwise *F*_ST_ between sampling locations from MAPI analysis.

We received a total of 1,373,095,567 reads for the *SbfI* dataset including 760 individuals. Following quality filtering, the initial alignment in Stacks (“gstacks” module) identified an average of 630,432 reads per individual (sd 232,000), including 303,315 variable sites at an average depth of 320.0× (sd 114.4×). Of these, Stacks *Populations* module retained 16,631 variable sites at r = 0.5. After final filtering of the VCF file output from *Populations*, we retained 625 individuals with 819 RAD loci and 7041 total variants with 25% missing data. For analysis, we randomly sampled one SNP per RAD locus for a total of 819 unlinked SNPs. This final SbfI dataset contained individuals from 62 sampling locations (Figure 1).

We received 540,486,110 total reads from 380 individuals for the *PstI* sequencing dataset. The Stacks *Populations* module retained 16,528 RAD loci with *r* = 0.5 at an average coverage of 17×. Further filtering of the VCF reduced the final dataset to 2699 unlinked SNPs among 366 individuals from 34 sampling locations, with 19% missing data.

### 3.2 Genetic Diversity

For the *SbfI* dataset, nucleotide diversity (π) calculated in Stacks *Populations* module across all variant sites (*n* = 16,631) averaged 0.061 within sampling locations, ranging from 0.034 to 0.072 (SD ± 0.005; see Appendix S2). Expected heterozygosity (H_e_) averaged 0.056 (SD ± 0.007), with a range from 0.017 to 0.066. Observed heterozygosity (H_o_) was lower, averaging 0.031 among sampling locations. For the filtered dataset of 819 unlinked SNPs, H_e_ was higher, with a mean of 0.118 (SD ± 0.051). An AMOVA conducted on the filtered dataset partitioned 12.48% of the total genetic variation between sampling locations (Φ_ST_ = 0.125). Pairwise population *F*_ST_ statistics showed moderate differentiation with an overall average *F*_ST_ = 0.123 (±0.082 SD) for the filtered data.

Nucleotide diversity for the *PstI* dataset calculated in Stacks *Populations* module for all variant sites (*n* = 209,016) averaged 0.060, ranging from 0.038 to 0.076 (SD ± 0.007). Expected heterozygosity (H_e_) averaged 0.056 (SD ± 0.008), ranging from 0.019 to 0.072, and was higher than the average observed heterozygosity (H_o_) of 0.033. H_e_ values for the filtered dataset of 2699 unlinked SNPs were higher, with a mean of 0.282 (±0.042 SD). An AMOVA conducted on the filtered dataset of 2699 SNPs partitioned 11.50% of the total genetic variation between sampling locations (Φ_ST_ = 0.115). Pairwise population *F*_ST_ statistics had an overall average *F*_ST_ = 0.09 (± 0.053 SD), indicating moderate differentiation.

### 3.3 Population Structure

We found largely the same pattern of genetic population structure across ancestry analyses, regardless of method or dataset (Figure 1A; Appendix S3). STRUCTURE analyses conducted on the *SbfI* dataset showed a peak Δ*K* = 2, splitting the western and eastern Mojave Desert sampling locations along a longitudinal gradient. However, both the log probabilities of the data and ΔK indicated further population structuring, with a secondary peak at *K* = 4. This solution segregated sampling locations into four geographically distinct populations in the northwest, southwest, northeast, and southeast quadrants of the Mojave Desert ecoregion (Figure 1), with some admixture apparent in transitional areas between each population. The extent of these transitional areas was estimated in the POPMAPS spatial interpolation of ancestry coefficients, which showed the most uncertainty in population assignment (e.g., broadest transitional area) between the northwest and southwest populations (Figure 1B). DAPC conducted on the *SbfI* dataset also indicated *K* = 4 as the solution with optimal BIC and mapped sampling locations to nearly the same populations as STRUCTURE, although with less admixture apparent in the transition zones (Appendix S3). DAPC of the *PstI* data showed a strikingly similar pattern, with the same population groups in the northwest, southwest, and southeast (Appendix S3). Populations from the northeast were not available in this dataset, and BIC therefore indicated *K* = 3 as the optimal solution. The MAPI interpolation of pairwise *F*_ST_ values between sampling locations gave further support for the genetic population divisions from ancestry analyses, with the highest differentiation apparent between the western and eastern populations, particularly for the northeast group (Figure 1C).

### 3.4 Genome Scans for Selection

**FIGURE 2:**
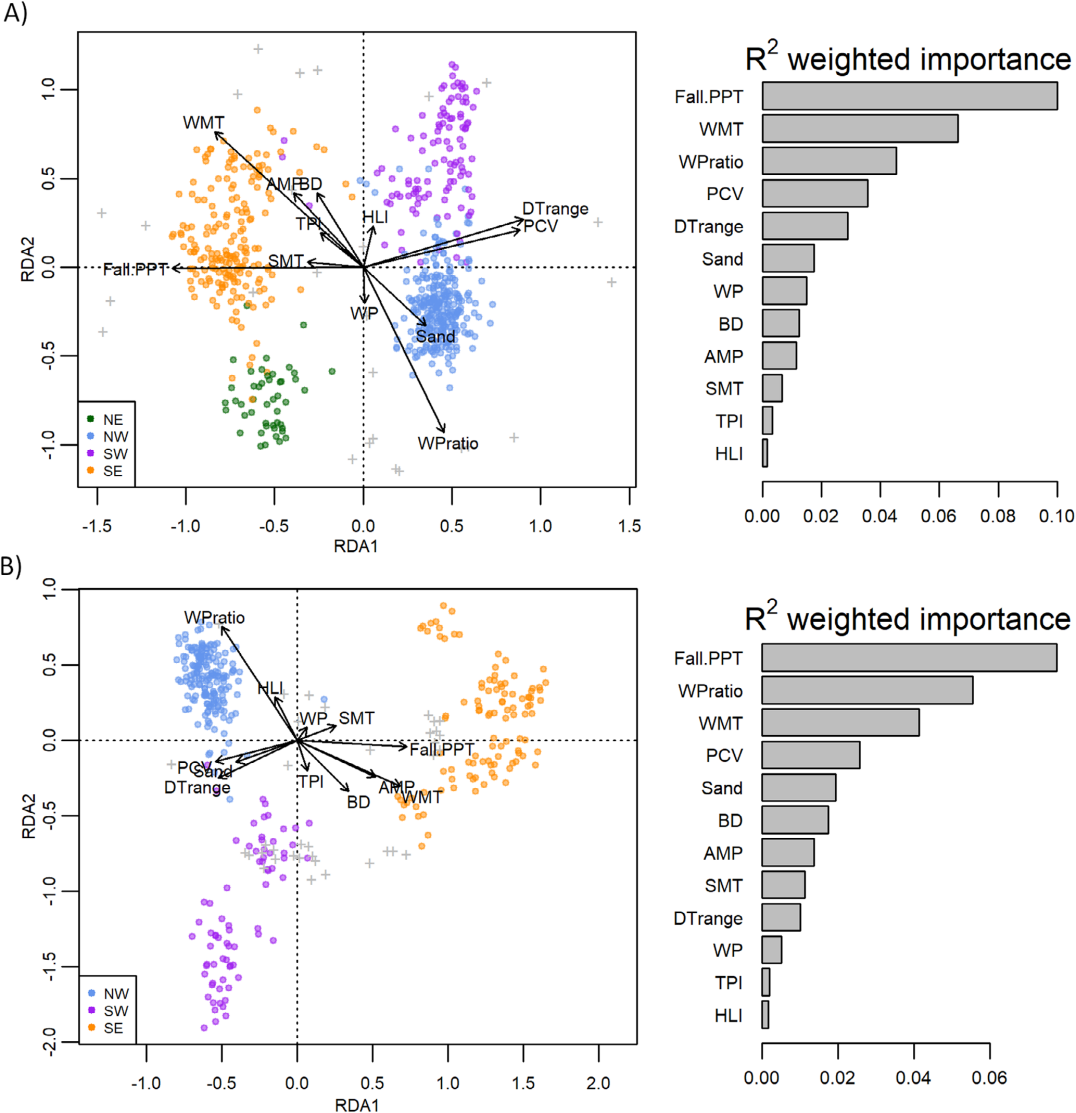
*Left panels*: RDA biplots from genome scans for selection on the (A) *SbfI* and (B) *PstI* datasets. Arrows indicate the strength and direction of associations between allele frequencies and environmental variables. Colors within the biplots correspond to the four genetic populations from ancestry analysis. Tick marks represent loci scores in the ordination. *Right panels*: GF importance plots showing environmental variables in order of relative *R* ^2^ weighted importance. See Table 1 for variable codes.

We detected 86 potentially adaptive loci within the *PstI* dataset (approximately 3% of the 2699 SNPs) and 44 candidate loci from the *SbfI* dataset (5.4% of 819 SNPs) based on three significant RDA axes for each dataset, paired with the BayeScEnv runs (Appendix S4). BayeScEnv identified 20 potentially adaptive loci from the SbfI dataset and 61 potentially adaptive loci from the PstI dataset, while RDA identified 26 and 36 potentially adaptive loci from these datasets, respectively (Appendix S4). The larger proportion of potentially adaptive loci in the *SbfI* dataset may be explained by a larger sample size of populations (*n* = 62 vs. *n* = 34 for the *PstI* data), which might better resolve genetic variation along environmental gradients. RDA biplots indicated that the overall genetic structure and influence of environmental variables were similar across datasets (Figure 2A,B for *PstI* and *SbfI* datasets, respectively), with winter precipitation ratio (WPratio), winter minimum temperature (WMT), fall precipitation (Fall.PPT), and diurnal temperature range (DTrange) having the most influence on RDA axes. Genetic population structure was also apparent in the biplots, suggesting that differences in influential environmental gradients correspond to the different geographic divisions of populations. GF models fit the potentially adaptive loci frequencies corresponded to RDA and ranked the same three variables as highest in permuted *R* ^2^ importance, including Fall.PPT (highest across both datasets), WMT, and WPratio (Figure 2A,B, right panels).

### 3.5 Resistance Surface Modeling

Mantel correlations were high for both geographic distance (IBD) and habitat resistance distance (IBR) across both neutral and potentially adaptive loci (Table 2). However, the correlation between geographic and genetic distance was eliminated by the partial Mantel test with habitat resistance. In contrast, the partial Mantel correlation for habitat resistance remained high even when geographic distance was removed. Mantel correlations with environmental distance were lower for both neutral and potentially adaptive loci but followed a pattern that would be expected under local adaptation, with a higher environmental correlation for potentially adaptive loci that remained positive in the partial Mantel tests. Our results in aggregate suggest IBR followed by local adaptation within suitable habitat. This interpretation is supported by the RDA analysis (see above), which showed that environmental gradients associated with outlier loci were parallel to the overall population divisions from ancestry analysis (see Figures 1 and 2).

**TABLE 2:** Mantel and partial Mantel tests quantifying associations among genetic, environmental, geographic, and habitat resistance distances for the *SbfI* dataset.

Scenario	Mantel test	Neutral Loci		Potentially Adaptive Loci	
		r	*p*	r	*p*
IBD	Genetic × Geographic	0.458	0.000	0.612	0.000
	Genetic × Geographic (− Habitat resistance)	−0.19	0.999	−0.19	0.999
	Genetic × Geographic (− Environment)	0.448	0.000	0.563	0.000
IBR	Genetic × Habitat resistance	0.506	0.000	0.644	0.000
	Genetic × Habitat resistance (− Environment)	0.499	0.000	0.612	0.000
	Genetic × Habitat resistance (− Geographic)	0.305	0.000	0.360	0.000
IBE	Genetic × Environment	0.106	0.06	0.281	0.000
	Genetic × Environment (− Habitat resistance)	−0.05	0.77	0.125	0.015
	Genetic × Environment (− Geographic)	−0.01	0.58	0.164	0.002

*Note:* Test results are contrasted between datasets of neutral and potentially adaptive genetic loci.

### 3.6 Multivariate Models of Potentially Adaptive Loci

**FIGURE 3:**
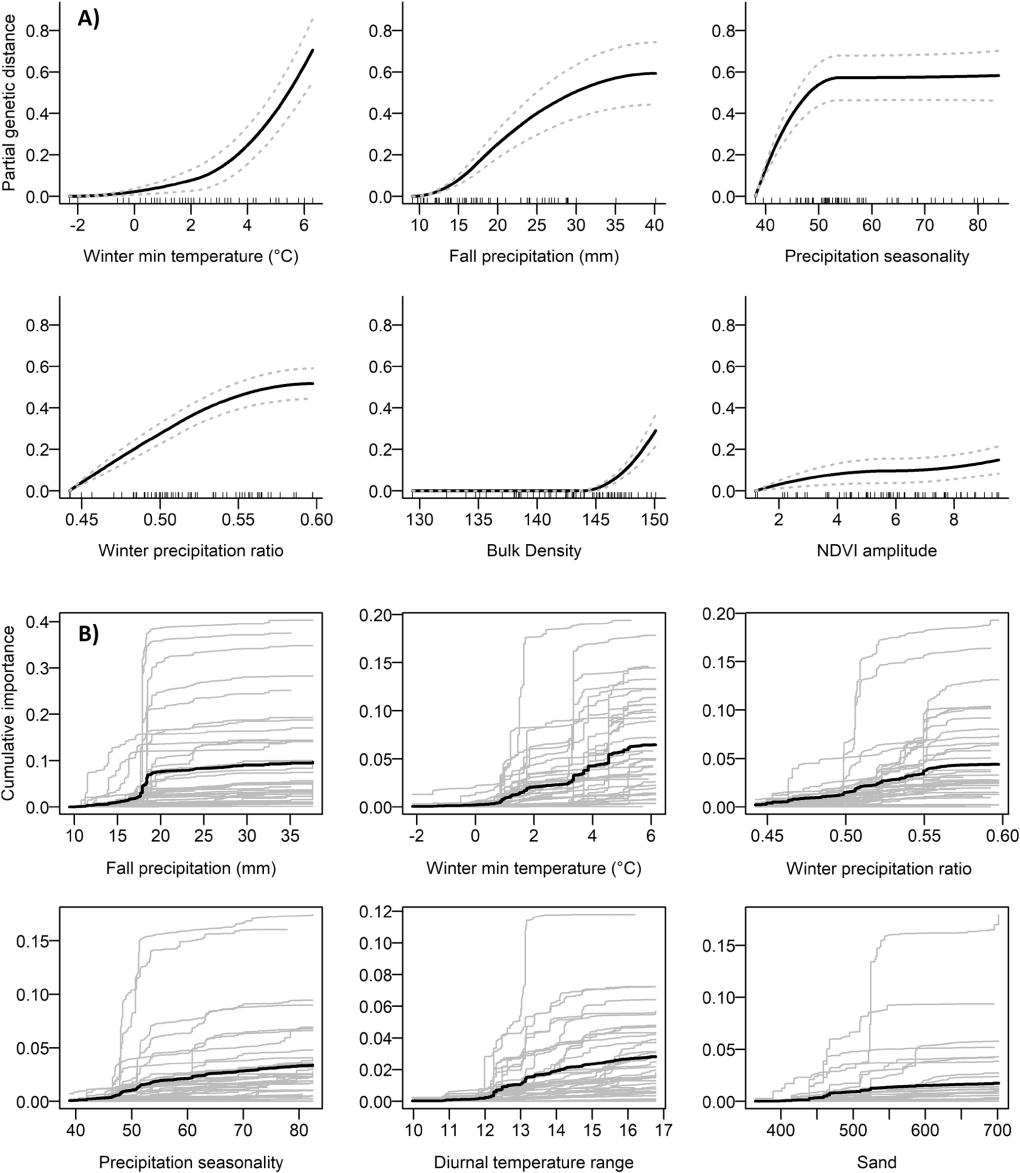
Multivariate models of potentially adaptive allele frequencies for the *SbfI* dataset. (A) Monotonic I‐splines from GDM. The height of each spline indicates the total amount of allele frequency turnover explained by each predictor, while the slope of each spline indicates the rate of change in allele frequencies along the gradient. Dashed lines indicate standard deviations derived from bootstrapping the GDM models with 999 permutations. (B) Cumulative turnover functions from GF for each environmental predictor (with individual SNP functions shown in light gray). The height of each function indicates variable importance, with “steps” in each curve denoting variable splits of high‐weighted *R* ^2^ importance along each gradient.

When including environmental variables alone, GDM and GF models indicated similar variable importance and response functions of potentially adaptive loci along environmental gradients for the *SbfI* dataset. The GDM including only environmental variables explained 33.56% of the deviance in allele frequencies and selected WMT, Fall.PPT, and PCV as the variables explaining the most genetic turnover (Figure 3A). The I‐spline for WMT suggested a threshold near 2°C where allele frequencies rapidly change. Similarly, the I‐spline for PCV showed a threshold between 40% and 50%, distinguishing areas with more seasonal precipitation variability (generally corresponding to summer/fall precipitation). The GF model explained approximately 33.58% of the variation in potentially adaptive allele frequencies, with Fall.PPT, WMT, and WPratio showing highest permuted importance (Figure 3B). The allele turnover functions from this method indicated a sharp threshold in Fall.PPT at approximately 17 mm, along with a threshold in WMT between 1°C and 2°C similar to the GDM spline. GF also indicated that potentially adaptive allele frequencies were associated with WPratio, showing a threshold between 0.50% and 0.55%. Overall, environmental response functions from GDM and GF were highly correlated with each other: Linear regressions showed *R* ^2^ values of 0.935 for Fall.PPT, 0.895 for WMT, 0.922 for WPratio, and 0.456 for PCV when GDM and GF response functions were compared (see Appendix S5 for spatial comparison). GF and GDM curves for the *PstI* dataset were markedly similar and are provided in the supplement (Appendix S6). Notably, the GDM model for the *PstI* dataset selected the same six most important variables while explaining 42% of the deviance in allele frequencies.

A comparison of alternative GDM models on the *SbfI* dataset including geographic distance and habitat resistance, in addition to environmental distance, found that a GDM incorporating environmental distance and habitat resistance (pairwise least‐cost habitat resistance matrix) gave the best overall fit, explaining 56.06% of the deviance in potentially adaptive allele frequencies (Appendix S7). WMT, PCV, WPratio, and Fall.PPT were again the top environmental terms in this model, while habitat resistance was the most important overall predictor. As with the partial Mantel tests, geographic distance was not selected in the GDM model when habitat resistance was included, suggesting that habitat resistance better explains genetic variation across the landscape.

### 3.7 Seed Transfer Zones and Future Projection

**FIGURE 4:**
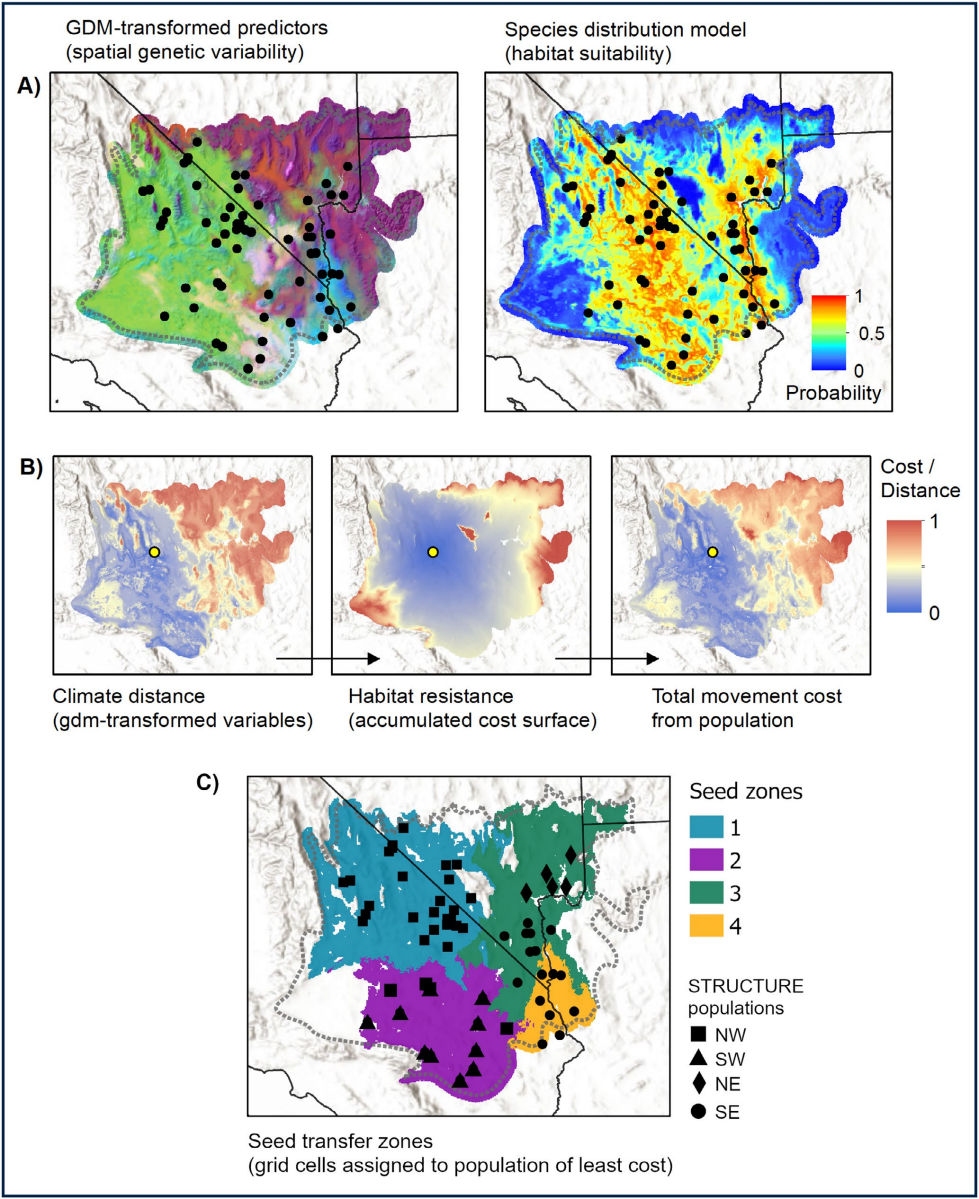
Flowchart of seed transfer zone delineation analysis. (A) Inputs include GDM‐transformed environmental predictors, reflecting gene–environment associations, here displayed as an RGB composite where similar colors indicate similar genetic composition; and a species distribution model (SDM) with probabilities of occurrence. (B) Seed zones are based on accumulated movement cost from population centers (here, represented by the yellow point in the panels), including genetic distance (multivariate Euclidean distance on GDM‐transformed environmental predictors) and habitat resistance (least cost paths from SDM). The two layers are combined to reflect total movement cost. (C) A nearest‐neighbor search is used to assign grid cells to the population of least total movement cost, resulting in seed transfer zones.

GDM‐transformed environmental predictors, in combination with least‐cost habitat resistance, were used to assign grid cells to seed transfer zones within the Mojave Desert ecoregion (Figure 4A,B). Cluster silhouette statistics from the hierarchical agglomerative cluster analysis of GDM‐predicted genetic distances showed a peak at a dendrogram cut point of *k* = 4 (S_(i)_ = 0.38), suggesting four seed transfer zones best balanced the within‐ and between‐cluster genetic variation. The four resulting groups explained 64.47% of the variation in the GDM‐predicted genetic distances based on perMANOVA. Even though the GDM was based only on potentially adaptive loci, the resulting seed transfer zones largely mirrored the four genetic populations, differing slightly in the east where a portion of the sampling locations within the broader southeast genetic population were contained within the same seed transfer zone as those of the northeast genetic population (Figure 4C, right panel). For management purposes, we also calculated a version of the seed transfer zones with six zones (Appendix S8), which explained 73.24% of the GDM predicted genetic distances but had a lower silhouette statistic (*S*_(i)_ = 0.33).

**FIGURE 5:**
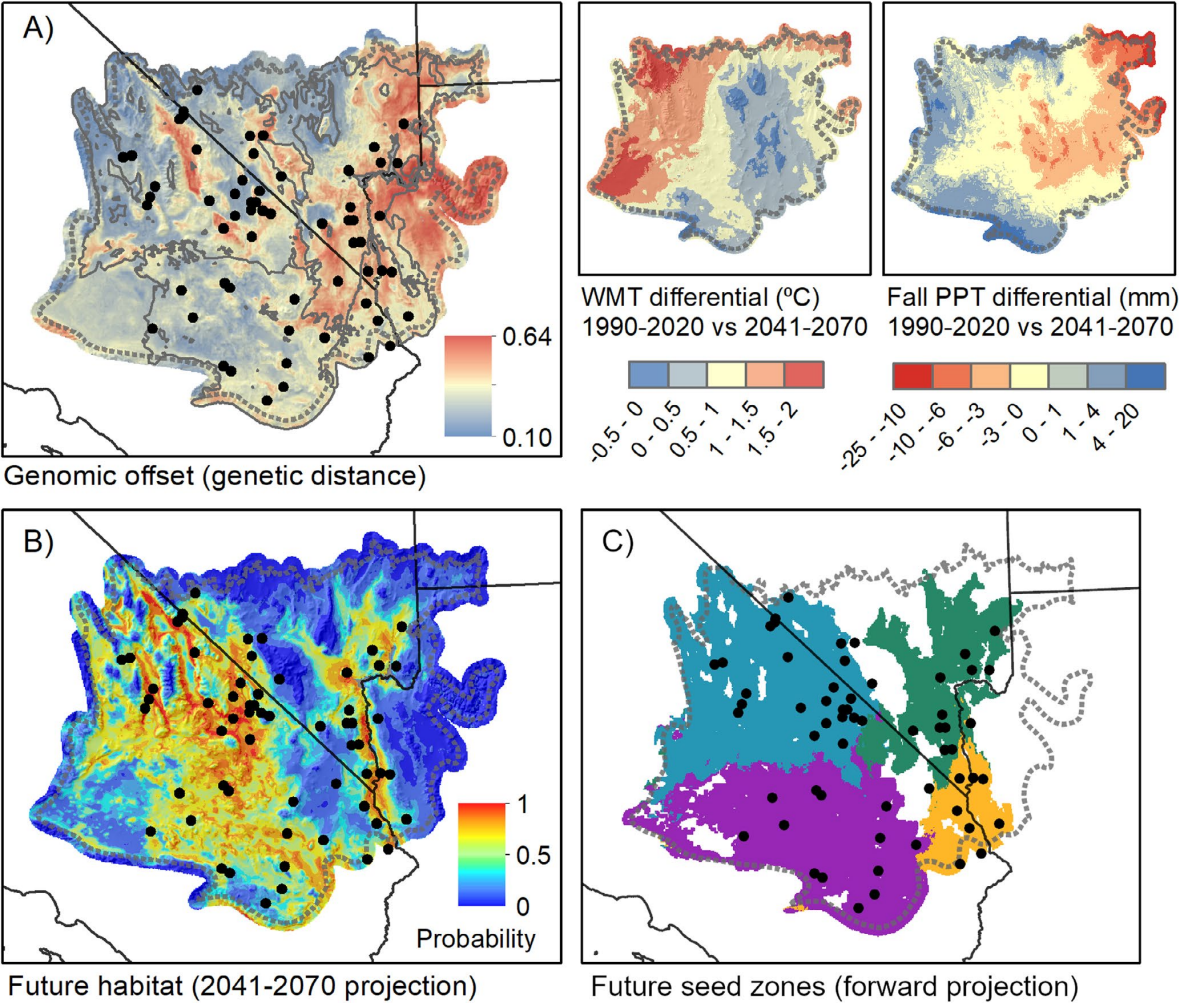
(A) Genomic offset predicted from GDM indicating areas most susceptible to climate change, where rapid changes in allele frequencies would be necessary to track changes in climate based on the modeled environmental associations. The offset is largely due to predicted changes in winter minimum temperature and fall precipitation (right panels). (B) A forward‐predicted species distribution model (SDM) of *Chylismia brevipes* habitat for the period 2041–2070 is used to project seed transfer zones into future climate space (C).

Genomic offset for both the *SbfI* and *PstI* datasets was most pronounced in the east (Figure 5A; Appendix S9), associated with a predicted decline in fall precipitation for that area (Figure 5A), leading to an increase in PCV. The change in precipitation regime was also reflected by a reduction in suitable habitat in the east projected by the future‐climate SDM (Figure 5B). Populations in the western Mojave Desert generally had lower genomic offset than those from the east. Higher winter temperatures, coupled with an increase in fall precipitation (Figure 5A), shifted habitat more favorably to the west (Figure 5B), which was also reflected in the future‐projected seed transfer zones (Figure 5C). Consequently, the area of unsuitable habitat and genetic discontinuity dividing eastern and western populations is predicted to expand relative to current conditions (Figures 4A,C and 5B,C).

## 4 Discussion

Understanding relationships among genotypes, their environments, and genetic population structure is critical to managing native populations and promoting resilient native plant communities through habitat restoration. Genome scans have given practitioners a cost‐efficient mechanism for obtaining knowledge of potentially adaptive genetic variation along environmental gradients from high‐priority restoration species. Here, we demonstrated that key environmental associations, as well as genetic population structure, were largely consistent across two relatively low‐density RAD‐seq datasets generated using different restriction enzymes, and, consequently, at different marker densities. In particular, we found that several key environmental gradients, including fall precipitation, WMT, and the monthly distribution of precipitation in winter, were most strongly associated with potentially adaptive loci regardless of analysis or dataset. Both the *PstI* dataset (with 2600 SNPs) and the *SbfI* dataset (with 819 SNPs) provided comparable insights for management of our study species.

One caveat to our approach is that, unlike common garden experiments (Custer et al. 2022), genome scans for selection typically do not provide information on the functional relevance of potentially adaptive loci or their effect on plant fitness. Moreover, genome scans can be confounded by neutral genetic processes, such as founder effects, population bottlenecks, and IBD (Excoffier, Hofer, and Foll 2009; Schoville et al. 2012). While our analyses were able to exclude the latter as the most likely explanation for the genetic structure we observed, we still found that neutral and putatively adaptive genetic variation segregated along parallel spatial gradients, making these processes difficult to disentangle. However, concordance of overall genetic structure with patterns of habitat connectivity and resistance, rather than geographic distance, suggests that IBE is a large influence, resulting from the restriction of gene flow within connected patches of suitable habitat due to limits on pollination and seed dispersal. IBE can substantially influence the genome by elevating genomic islands of divergence (Nosil, Funk, and Ortiz‐Barrientos 2009), thereby increasing the signal of selection detectable in genome scans (Yoder and Tiffin 2018), depending on the strength of linkage and/or divergence hitchhiking (e.g., Larson et al. 2017). Consequently, strong but selectively neutral genetic differentiation may become associated with the same environmental gradients as adaptive variation under IBE, and both processes would tend to increase genetic incompatibilities with distance along environmental gradients (e.g., Massatti and Knowles 2020). Such a process could explain the concordant spatial pattern we observed between population subdivisions and environmental associations of potentially adaptive loci (Figure 2). For both datasets, we identified potentially adaptive loci linked to the same gradients—fall precipitation, WMT, and PCV (Figure 3). When translated into seed transfer zones, these environmental associations resulted in zones approximately the same as those from the POPMAPS analysis, which were based only on genetic ancestry and habitat resistance (Figure 1); in both cases, spatial patterns likely reflect historic patterns of genetic ancestry as well as divergence through isolation and local adaptation. Congruence of population structure, environmental gradients, and potentially adaptive loci may arise from the sharp topography of our study region, where extreme gradients in elevation, precipitation, and temperature both constrain dispersal for species outside of their habitat and drive desert species to evolve specific adaptations to their environments. While relatively few studies have assessed local adaptation in the Mojave Desert (but see Custer et al. 2022; Shryock et al. 2017, 2021), it is extremely common in the mountainous Desert Southwest (Baughman et al. 2019). Given that the winter‐rainfall dominated Mojave Desert climate emerged only recently in geologic terms (late Pleistocene; Thorne 1986), selection on standing genetic variation, driven by temporal environmental variation (Schemske and Bierzychudek 2001), could maintain adaptive divergence along contemporary climate/topographic gradients. At the same time, neutral genetic variation in the Mojave Desert has likely been shaped by a number of processes, including Pleistocene climate fluctuations causing habitat fragmentation and shifts in species distributions, as well as pre‐Pleistocene vicariance arising from inundation events in the Mojave and Sonoran Deserts (Wood et al. 2012).

While our methods do not allow us to verify the function of potentially adaptive loci, we note that the key environmental gradients identified across our datasets have obvious relevance for *C. brevipes'* biology and life history. Germination of winter annuals in the Mojave Desert is triggered by abundant rains in fall, or in late winter with warming winter temperatures (Beatley 1974); subsequent composition and/or biomass of annuals depends on the monthly distribution and amount of winter rainfall (Beatley 1969; Bowers 1987). A threshold of 15–25 mm of fall precipitation generally is known to trigger mass germination of winter annuals (Beatley 1974), and, interestingly, we found a corresponding threshold in potentially adaptive loci frequencies near this quantity of average fall precipitation (Figure 3). We also found that the distribution of winter rainfall and warmer WMT was associated with thresholds in potentially adaptive loci, distinguishing individuals from regions with a greater proportion of late winter rainfall. If accurate, these environmental associations could suggest that individuals are preadapted to respond to critical fall or late winter rain in regions with a higher frequency of such precipitation events. A similar adaptive cline was noted among populations of the sympatric winter annual, *Plantago ovata* (formerly *P. insularis* ), when grown in a common garden experiment (Clauss and Venable 2000): Populations varied in their ability to germinate in response to precipitation cues depending on the precipitation regime of the home environment. The strong association between potentially adaptive loci and WMT could reflect intraspecific variation in germination phenology or overwinter growth. Temperature conditions of the soil following fall and winter rains have a strong influence on germination response (Beatley 1974), and our data suggest there may be an allele frequency threshold where the WMT average exceeds 2°C–4°C. However, daytime temperatures during the winter months are also associated with plant growth and growth‐form development (Mulroy and Rundel 1977), and populations from warmer climates could favor earlier growth.

Genomic offset has received increased interest as a means of predicting potential maladaptation of genotypes to future climate (Capblancq et al. 2020; Fitzpatrick et al. 2021; Rellstab, Dauphin, and Exposito‐Alonso 2021), with limited evidence suggesting the technique may be effective in predicting population fitness following seed transfer (Fitzpatrick et al. 2021). Our approach to genomic offset is similar to previous efforts but incorporates a future‐projected SDM and least‐cost path analysis to project seed transfer zones into future climate space (Figure 5). Both the future‐projected seed transfer zones (Figure 5C) and genomic offset map (Figure 5A) indicated that eastern Mojave Desert populations may be at greater risk of disruption, in large part due to a decrease in fall precipitation predicted by the CMIP6 GCMs (Figure 5A, right inset). This trend in precipitation could shift germination to later in the growing season, when soil temperatures are lower than the range favorable for plant growth (Beatley 1974). Previous evidence suggests that a shift toward later winter precipitation could favor slower‐growing, cold‐adapted species or ecotypes among winter annuals (Kimball et al. 2010), shifting favorable habitat to the west and putting current eastern Mojave Desert populations at a disadvantage if they rely on fall precipitation, as has been noted for *Onagraceae* as well as other families (e.g., *Polemoniaceae* and *Polygonaceae*; Beatley 1974; Mulroy and Rundel 1977). A westward shift in habitat, however, requires synchrony between *C. brevipes* and its pollinators for this obligate outcrossing species, which could be decoupled if, for example, climate cues for flowering are offset from those that trigger emergence of adult pollinators from diapause (CaraDonna, Cunningham, and Iler 2018). A change in precipitation regime could also have unpredictable consequences for competitive interactions with invasive annual grasses, such as red brome ( *Bromus madritensis* ssp*. rubens* ), which competes effectively through early emergence and rapid acquisition of resources and may require less overall precipitation than native species to establish (Beatley 1966). Conversely, red brome does not exhibit adaptive bet hedging (i.e., seed bank dormancy), unlike most native winter annuals, rendering its populations more susceptible to an increase in drought frequency predicted for the Southwest (Dai 2013; DeFalco et al. 2003; Salo 2004). While difficult to predict, impacts of changing precipitation regime on populations and species composition of winter annuals in the Mojave Desert, and their interactions with invasives, could have cascading effects across trophic levels, as key herbivores such as the Mojave desert tortoise ( *Gopherus agassizii* ) rely on native forbs to maintain populations (Esque, Drake, and Nussear 2014) and are harmed by invasives species in their diets (Drake et al. 2016).

### 4.1 Management Implications

*C. brevipes* is a desirable species for restoration in the Mojave Desert based on its broad range, support for native pollinators, and use as forage by the Mojave desert tortoise and other wildlife species (Esque et al. 2021). Using genome scans, we find evidence that *C. brevipes* is locally adapted to fall and winter precipitation and temperature, and habitat restoration strategies involving this species may benefit by accounting for these differences in source population climates. Seed transfer zones, which delineate areas within which native plant materials may be transferred to limit risk of maladaptation, were identified to guide restoration treatments and seed collections for *C. brevipes* in the Mojave Desert (Figure 4; Appendix S8), including layers with four and six zones depending on the spatial refinement needed (Shryock, DeFalco, and Esque 2020; https://doi.org/10.5066/P9BQ6IYJ). Due to the pattern of IBR and/or IBE in *C. brevipes* , seed transfer zones identified here reflect patterns of both putatively adaptive and neutral genetic variation and broadly follow the inferred population genetic structure from ancestry analyses. Future field experiments could verify the functional relevance of climate associations detected in this work and their relevance for population fitness.

## Conflicts of Interest

The authors declare no conflicts of interest.

## Supporting information


Data S1.

## Data Availability

Genetic Data: DNA sequences have been deposited in the SRA under BioProject PRJNA1173917. VCF genotype files will be available from ScienceBase (https://doi.org/10.5066/P9AXJ18M). Environmental Data: Species habitat data are available from ScienceBase (https://doi.org/10.5066/P9XQJFEL). Seed transfer zones are available from ScienceBase (https://doi.org/10.5066/P9BQ6IYJ)
