# Supplementary material for: Concordant Signal of Genetic Variation Across Marker Densities in the Desert Annual Chylismia brevipes Is Linked With Timing of Winter Precipitation
Source: Evol Appl. 2024 Dec 16;17(12):e70046. doi: 10.1111/eva.70046 (PMC11649585; doi:10.1111/eva.70046)

**Supplemental Information for:**

**Concordant signal of genetic variation across marker densities in the desert annual *Chylismia brevipes* is linked with timing of winter precipitation**

**Daniel F. Shryock^1^, Nila Lê^2^, Lesley A. DeFalco^1^, and Todd C. Esque^1^**

**^1^U.S. Geological Survey, Western Ecological Research Center, 500 Date Street, Boulder City, NV**

**^2^California Botanic Garden, 1500 N. College Avenue, Claremont, CA 91711**

**Table of Contents:**

| **Appendix S1. Population locations and variables** | Page 2 |
| --- | --- |
| **Appendix S2. Population genetic summary statistics** | Page 4 |
| **Appendix S3. Additional population structure results** | Page 8 |
| **Appendix S4. List of potentially adaptive loci** | Page 12 |
| **Appendix S5. GF and GDM transformed predictors** | Page 16 |
| **Appendix S6. Additional gradient forest results** | Page 17 |
| **Appendix S7. Additional GDM results** | Page 19 |
| **Appendix S8. Seed transfer zones** | Page 20 |
| **Appendix S9. Genomic offset** | Page 22 |

**Appendix S1.**

Table 1. Populations UTM coordinates (NAD 83 UTM Zone 11N) and environmental variables extracted at population locations.

| Pop | Easting | Northing | AMP | DT | PCV | SMT | SP | WMT | WP | WPr | Fall.PPT | Sand | BD | Elev (m) |
| --- | --- | --- | --- | --- | --- | --- | --- | --- | --- | --- | --- | --- | --- | --- |
| AFNO | 551057 | 3883598 | 4.98 | 15.48 | 55.30 | 39.60 | 27.00 | 2.30 | 65.00 | 0.48 | 12.06 | 612.46 | 138.39 | 626 |
| AFTO | 556435 | 3877833 | 3.60 | 16.65 | 53.75 | 40.80 | 26.00 | 1.30 | 62.00 | 0.46 | 12.00 | 624.33 | 129.38 | 477 |
| AMRD | 618531 | 3795204 | 2.19 | 15.43 | 51.11 | 40.20 | 33.00 | 4.30 | 53.00 | 0.45 | 17.77 | 687.20 | 143.98 | 501 |
| BITB | 721421 | 4014833 | 4.45 | 13.21 | 45.94 | 40.50 | 53.00 | 3.30 | 95.00 | 0.52 | 26.53 | 618.20 | 146.84 | 622 |
| BMRD | 570165 | 3995775 | 5.78 | 15.08 | 58.14 | 37.90 | 37.00 | 0.40 | 90.00 | 0.56 | 14.80 | 616.09 | 141.15 | 957 |
| BOCA | 505692 | 4067222 | 3.66 | 14.53 | 55.84 | 37.50 | 37.00 | 0.60 | 85.00 | 0.58 | 13.95 | 586.16 | 146.05 | 1054 |
| CAGU | 659972 | 3823665 | 4.71 | 13.19 | 53.60 | 38.00 | 49.00 | 4.00 | 86.00 | 0.49 | 22.95 | 644.36 | 142.43 | 826 |
| CCRD | 503221 | 3875377 | 8.45 | 13.69 | 67.53 | 36.80 | 31.00 | 3.40 | 112.00 | 0.49 | 14.00 | 703.28 | 146.37 | 917 |
| CCWA | 739867 | 4014602 | 8.24 | 13.83 | 38.08 | 40.60 | 65.00 | 2.90 | 90.00 | 0.50 | 28.96 | 586.72 | 145.61 | 613 |
| CHVL | 580042 | 3971717 | 5.28 | 15.67 | 62.87 | 39.90 | 36.00 | 0.80 | 97.00 | 0.55 | 14.82 | 563.18 | 143.13 | 632 |
| CHWA | 731571 | 3814711 | 6.66 | 15.29 | 51.98 | 42.80 | 41.00 | 4.80 | 70.00 | 0.49 | 19.00 | 430.00 | 142.50 | 244 |
| COTC | 691561 | 3927318 | 8.69 | 11.90 | 47.92 | 36.70 | 61.00 | 3.40 | 101.00 | 0.51 | 30.00 | 699.98 | 147.17 | 1047 |
| CPRK | 516129 | 3845482 | 9.46 | 14.24 | 73.51 | 34.00 | 39.00 | 2.50 | 158.00 | 0.53 | 15.54 | 631.94 | 145.95 | 1201 |
| CRST | 578232 | 4044098 | 3.71 | 16.90 | 51.51 | 39.00 | 36.00 | -0.60 | 74.00 | 0.54 | 15.84 | 633.21 | 145.32 | 811 |
| DAPA | 506178 | 4071181 | 9.47 | 14.17 | 53.26 | 35.20 | 43.00 | -0.60 | 97.00 | 0.57 | 16.00 | 640.67 | 142.61 | 1333 |
| DENS | 549109 | 3942901 | 2.58 | 16.28 | 54.94 | 42.60 | 26.00 | 2.00 | 59.00 | 0.55 | 12.53 | 551.92 | 132.28 | 281 |
| DEVA | 449136 | 4023221 | 5.01 | 12.83 | 68.67 | 34.70 | 36.00 | 1.30 | 121.00 | 0.56 | 12.47 | 670.21 | 147.53 | 1341 |
| DUGU | 562092 | 3980947 | 4.23 | 15.17 | 71.21 | 39.40 | 34.00 | 0.90 | 100.00 | 0.59 | 13.36 | 577.95 | 140.65 | 737 |
| DVBT | 501227 | 4062739 | 2.88 | 15.03 | 53.50 | 41.70 | 27.00 | 3.00 | 61.00 | 0.57 | 9.11 | 510.70 | 134.74 | 573 |
| EDJR | 719866 | 3956774 | 7.65 | 11.71 | 39.55 | 38.10 | 91.00 | 4.20 | 107.00 | 0.50 | 40.14 | 627.31 | 146.96 | 940 |
| ELDO | 691925 | 3952106 | 5.75 | 11.75 | 50.53 | 35.00 | 68.00 | 1.80 | 117.00 | 0.50 | 26.00 | 604.67 | 141.00 | 1291 |
| FRAN | 751053 | 3846904 | 6.31 | 15.61 | 51.50 | 41.70 | 56.00 | 4.00 | 92.00 | 0.54 | 23.75 | 454.98 | 148.61 | 355 |
| GAWA | 708136 | 3802646 | 7.67 | 13.62 | 58.87 | 40.20 | 46.00 | 5.10 | 79.00 | 0.50 | 20.00 | 507.67 | 148.00 | 518 |
| GCRD | 614853 | 3770049 | 5.04 | 15.98 | 51.20 | 39.60 | 33.00 | 3.40 | 54.00 | 0.44 | 17.34 | 534.85 | 148.01 | 558 |
| GFBA | 465900 | 3968490 | 7.34 | 13.06 | 80.99 | 38.50 | 27.00 | 4.30 | 102.00 | 0.51 | 10.51 | 513.79 | 141.61 | 832 |
| H160 | 582334 | 4024922 | 6.11 | 15.15 | 48.12 | 37.50 | 43.00 | 0.20 | 92.00 | 0.56 | 16.93 | 647.57 | 144.82 | 964 |
| HITW | 519908 | 4028981 | 2.13 | 14.94 | 54.97 | 42.90 | 25.00 | 4.00 | 61.00 | 0.55 | 10.59 | 472.10 | 135.11 | 326 |
| HRDD | 472105 | 3833419 | 8.73 | 15.54 | 77.07 | 35.60 | 26.00 | 1.60 | 115.00 | 0.51 | 12.23 | 688.01 | 149.33 | 966 |
| JUSP | 746373 | 4057374 | 6.78 | 13.14 | 41.10 | 39.20 | 53.00 | 3.00 | 83.00 | 0.52 | 24.00 | 630.43 | 143.29 | 713 |
| JUWA | 534014 | 3973679 | 2.96 | 16.28 | 75.06 | 44.10 | 18.00 | 3.20 | 63.00 | 0.60 | 9.79 | 448.05 | 133.93 | 175 |
| KECN | 686195 | 3952151 | 4.43 | 13.80 | 47.57 | 38.70 | 52.00 | 2.10 | 84.00 | 0.48 | 22.91 | 612.64 | 147.08 | 807 |
| KIEX | 601076 | 3959299 | 9.29 | 13.15 | 48.82 | 34.50 | 66.00 | 0.20 | 132.00 | 0.50 | 28.83 | 633.23 | 143.00 | 1364 |
| KIWE | 592280 | 3962590 | 6.73 | 13.74 | 53.72 | 36.60 | 52.00 | 0.70 | 119.00 | 0.51 | 23.72 | 641.72 | 145.03 | 999 |
| LAL1 | 707488 | 3896606 | 5.56 | 9.88 | 48.98 | 37.00 | 57.00 | 5.80 | 101.00 | 0.50 | 25.00 | 634.06 | 140.20 | 947 |
| LALN | 707432 | 3896410 | 8.22 | 10.07 | 48.92 | 37.70 | 57.00 | 6.00 | 99.00 | 0.50 | 25.00 | 614.83 | 139.19 | 861 |
| LAME | 722768 | 3897123 | 9.52 | 14.58 | 58.43 | 43.40 | 42.00 | 6.30 | 95.00 | 0.55 | 19.92 | 462.43 | 139.68 | 246 |
| LAVA | 674682 | 3885820 | 7.77 | 12.99 | 46.64 | 35.60 | 65.00 | 1.90 | 107.00 | 0.49 | 28.86 | 631.74 | 149.35 | 1137 |
| MEDA | 699126 | 3928391 | 5.13 | 12.95 | 47.49 | 39.40 | 50.00 | 4.00 | 85.00 | 0.50 | 24.10 | 621.60 | 144.70 | 706 |
| NEDI | 549299 | 3792573 | 9.03 | 15.43 | 83.90 | 35.10 | 30.00 | 1.70 | 138.00 | 0.52 | 12.54 | 623.36 | 138.04 | 1111 |
| NELA | 688500 | 3965908 | 5.50 | 13.81 | 42.73 | 39.90 | 46.00 | 2.70 | 72.00 | 0.50 | 21.01 | 586.50 | 146.10 | 655 |
| NOPE | 588939 | 3983514 | 7.27 | 16.08 | 68.57 | 38.40 | 32.00 | -0.40 | 102.00 | 0.56 | 12.19 | 630.16 | 145.18 | 840 |
| NOSO | 579562 | 3963768 | 6.05 | 15.43 | 64.71 | 39.70 | 36.00 | 1.10 | 100.00 | 0.55 | 15.09 | 591.41 | 146.59 | 700 |
| NPPA | 584982 | 3971522 | 5.69 | 15.08 | 57.64 | 38.30 | 42.00 | 0.60 | 104.00 | 0.54 | 17.22 | 596.54 | 143.08 | 864 |
| PIBA | 596907 | 3754613 | 4.06 | 16.44 | 53.67 | 37.80 | 36.00 | 1.90 | 65.00 | 0.48 | 16.65 | 606.48 | 149.70 | 751 |
| PINR | 520881 | 4094074 | 6.67 | 15.59 | 51.82 | 35.40 | 51.00 | -2.30 | 111.00 | 0.60 | 15.79 | 642.93 | 144.02 | 1298 |
| RAGD | 686592 | 4006509 | 1.25 | 15.47 | 44.63 | 41.50 | 38.00 | 1.80 | 67.00 | 0.51 | 18.00 | 615.68 | 138.49 | 535 |
| SACM | 704939 | 3861289 | 5.78 | 14.20 | 50.81 | 41.40 | 49.00 | 4.80 | 81.00 | 0.48 | 27.21 | 347.83 | 147.39 | 401 |
| SEPA | 469747 | 3977519 | 6.92 | 12.55 | 79.25 | 38.50 | 26.00 | 5.00 | 101.00 | 0.53 | 10.17 | 574.04 | 142.61 | 810 |
| SLHL | 580400 | 3934072 | 4.30 | 16.59 | 52.61 | 42.50 | 26.00 | 1.40 | 59.00 | 0.56 | 13.59 | 674.01 | 138.21 | 333 |
| SMCM | 656959 | 3948585 | 6.05 | 13.11 | 57.26 | 35.80 | 63.00 | 1.10 | 107.00 | 0.44 | 18.80 | 645.33 | 145.35 | 1152 |
| SNCS | 718237 | 3841947 | 6.28 | 14.03 | 51.29 | 41.40 | 50.00 | 5.00 | 81.00 | 0.48 | 26.00 | 381.16 | 150.09 | 416 |
| SPHL | 562111 | 3956760 | 2.62 | 16.08 | 64.96 | 42.40 | 26.00 | 2.10 | 70.00 | 0.56 | 11.00 | 633.27 | 135.66 | 377 |
| STMI | 592801 | 4044876 | 5.82 | 13.57 | 45.72 | 35.00 | 91.00 | -0.20 | 104.00 | 0.56 | 35.06 | 623.46 | 146.55 | 1311 |
| TACM | 439698 | 4021047 | 4.48 | 14.33 | 71.93 | 32.30 | 43.00 | -2.00 | 148.00 | 0.57 | 13.69 | 629.97 | 147.81 | 1581 |
| THMB | 733285 | 3895696 | 8.08 | 12.08 | 53.15 | 38.80 | 60.00 | 5.40 | 112.00 | 0.52 | 27.70 | 637.67 | 137.02 | 734 |
| TRCN | 612068 | 3999780 | 5.91 | 13.58 | 51.66 | 35.90 | 67.00 | 0.60 | 131.00 | 0.59 | 26.84 | 641.20 | 143.70 | 1270 |
| TRNA | 474808 | 3989416 | 2.29 | 16.33 | 63.83 | 41.80 | 25.00 | 1.60 | 69.00 | 0.52 | 9.74 | 471.74 | 138.06 | 368 |
| VMRD | 627444 | 3865970 | 7.00 | 13.87 | 54.25 | 36.70 | 49.00 | 2.50 | 103.00 | 0.48 | 25.67 | 617.35 | 141.16 | 972 |
| VOFI | 713735 | 4032228 | 7.37 | 12.42 | 57.58 | 38.20 | 57.00 | 2.50 | 114.00 | 0.56 | 28.71 | 625.59 | 142.98 | 900 |
| WIPT | 621024 | 3831386 | 5.49 | 14.86 | 54.75 | 40.50 | 31.00 | 3.70 | 60.00 | 0.47 | 14.03 | 597.64 | 145.59 | 522 |
| YUME | 557691 | 3787735 | 7.71 | 15.86 | 79.90 | 35.40 | 39.00 | 1.70 | 140.00 | 0.51 | 16.63 | 644.44 | 146.29 | 1051 |

**Appendix S2.**

**Table S1.** Summary statistics for variant sites from the Stacks *Populations* module for 66 Mojave Desert populations of *Chylismia brevipes* genotyped using the *SbfI* restriction enzyme.

| **Pop** | **H_o_** | **Var** | **H_exp_** | **Var** | **H_obs_** | **Var** | **π** | **Var** | **F_IS_** | **Var** |
| --- | --- | --- | --- | --- | --- | --- | --- | --- | --- | --- |
| AFNO | 0.968 | 0.008 | 0.060 | 0.017 | 0.032 | 0.008 | 0.064 | 0.020 | 0.089 | 0.071 |
| AFTO | 0.969 | 0.012 | 0.053 | 0.018 | 0.031 | 0.012 | 0.061 | 0.024 | 0.059 | 0.057 |
| AMRD | 0.966 | 0.009 | 0.058 | 0.017 | 0.034 | 0.009 | 0.062 | 0.019 | 0.077 | 0.062 |
| BITB | 0.966 | 0.008 | 0.062 | 0.017 | 0.034 | 0.008 | 0.066 | 0.020 | 0.089 | 0.071 |
| BMRD | 0.966 | 0.009 | 0.059 | 0.017 | 0.034 | 0.009 | 0.063 | 0.019 | 0.078 | 0.065 |
| BOCA | 0.968 | 0.008 | 0.056 | 0.016 | 0.032 | 0.008 | 0.060 | 0.019 | 0.077 | 0.063 |
| CAGU | 0.966 | 0.013 | 0.051 | 0.017 | 0.034 | 0.013 | 0.059 | 0.023 | 0.049 | 0.048 |
| CCRD | 0.971 | 0.007 | 0.055 | 0.016 | 0.029 | 0.007 | 0.059 | 0.019 | 0.081 | 0.067 |
| CCWA | 0.964 | 0.009 | 0.054 | 0.015 | 0.036 | 0.009 | 0.058 | 0.018 | 0.060 | 0.051 |
| CHVL | 0.966 | 0.009 | 0.052 | 0.015 | 0.034 | 0.009 | 0.056 | 0.017 | 0.060 | 0.051 |
| CHWA | 0.972 | 0.008 | 0.052 | 0.016 | 0.028 | 0.008 | 0.056 | 0.018 | 0.073 | 0.061 |
| CIMA | 0.970 | 0.009 | 0.046 | 0.015 | 0.030 | 0.009 | 0.050 | 0.017 | 0.047 | 0.043 |
| COCP | 0.973 | 0.007 | 0.049 | 0.015 | 0.027 | 0.007 | 0.053 | 0.017 | 0.069 | 0.058 |
| COTC | 0.970 | 0.024 | 0.027 | 0.012 | 0.030 | 0.024 | 0.042 | 0.031 | 0.018 | 0.019 |
| COTV | 0.971 | 0.008 | 0.053 | 0.016 | 0.029 | 0.008 | 0.057 | 0.018 | 0.074 | 0.062 |
| CPRK | 0.972 | 0.007 | 0.056 | 0.016 | 0.028 | 0.007 | 0.060 | 0.018 | 0.093 | 0.076 |
| CRST | 0.968 | 0.008 | 0.057 | 0.016 | 0.032 | 0.008 | 0.061 | 0.019 | 0.079 | 0.065 |
| DAPA | 0.966 | 0.033 | 0.017 | 0.008 | 0.034 | 0.033 | 0.034 | 0.033 | 0.000 | 0.000 |
| DENS | 0.967 | 0.008 | 0.060 | 0.017 | 0.033 | 0.008 | 0.063 | 0.019 | 0.086 | 0.070 |
| DEVA | 0.971 | 0.007 | 0.057 | 0.017 | 0.029 | 0.007 | 0.061 | 0.019 | 0.085 | 0.069 |
| DNWR | 0.974 | 0.007 | 0.051 | 0.015 | 0.026 | 0.007 | 0.055 | 0.017 | 0.076 | 0.064 |
| DUGU | 0.968 | 0.008 | 0.059 | 0.017 | 0.032 | 0.008 | 0.063 | 0.019 | 0.086 | 0.070 |
| DVBT | 0.967 | 0.008 | 0.058 | 0.017 | 0.033 | 0.008 | 0.061 | 0.019 | 0.080 | 0.065 |
| EDJR | 0.966 | 0.009 | 0.061 | 0.017 | 0.034 | 0.009 | 0.065 | 0.020 | 0.086 | 0.069 |
| ELDO | 0.972 | 0.007 | 0.059 | 0.017 | 0.028 | 0.007 | 0.063 | 0.020 | 0.097 | 0.078 |
| FRAN | 0.968 | 0.008 | 0.056 | 0.016 | 0.032 | 0.008 | 0.059 | 0.019 | 0.076 | 0.061 |
| GAWA | 0.967 | 0.009 | 0.057 | 0.017 | 0.033 | 0.009 | 0.061 | 0.020 | 0.076 | 0.062 |
| GCRD | 0.973 | 0.007 | 0.052 | 0.016 | 0.027 | 0.007 | 0.056 | 0.018 | 0.079 | 0.065 |
| GFBA | 0.972 | 0.007 | 0.057 | 0.017 | 0.028 | 0.007 | 0.061 | 0.019 | 0.090 | 0.072 |
| H160 | 0.970 | 0.008 | 0.054 | 0.016 | 0.030 | 0.008 | 0.058 | 0.018 | 0.078 | 0.066 |
| HITW | 0.969 | 0.008 | 0.058 | 0.017 | 0.031 | 0.008 | 0.062 | 0.019 | 0.088 | 0.072 |
| HRDD | 0.974 | 0.007 | 0.053 | 0.016 | 0.026 | 0.007 | 0.056 | 0.018 | 0.079 | 0.066 |
| JUSP | 0.974 | 0.006 | 0.057 | 0.016 | 0.026 | 0.006 | 0.061 | 0.019 | 0.096 | 0.078 |
| JUWA | 0.968 | 0.011 | 0.056 | 0.018 | 0.032 | 0.011 | 0.064 | 0.024 | 0.068 | 0.063 |
| KECN | 0.972 | 0.007 | 0.057 | 0.017 | 0.028 | 0.007 | 0.062 | 0.020 | 0.089 | 0.075 |
| KIEX | 0.972 | 0.006 | 0.060 | 0.017 | 0.028 | 0.006 | 0.064 | 0.020 | 0.099 | 0.079 |
| KIWE | 0.969 | 0.007 | 0.056 | 0.016 | 0.031 | 0.007 | 0.060 | 0.018 | 0.079 | 0.065 |
| LAL1 | 0.972 | 0.007 | 0.059 | 0.017 | 0.028 | 0.007 | 0.064 | 0.019 | 0.099 | 0.080 |
| LALN | 0.972 | 0.007 | 0.060 | 0.017 | 0.028 | 0.007 | 0.064 | 0.020 | 0.101 | 0.081 |
| LAME | 0.970 | 0.007 | 0.058 | 0.017 | 0.030 | 0.007 | 0.062 | 0.019 | 0.088 | 0.072 |
| LAVA | 0.966 | 0.009 | 0.061 | 0.018 | 0.034 | 0.009 | 0.065 | 0.020 | 0.082 | 0.067 |
| LSCA | 0.972 | 0.007 | 0.054 | 0.016 | 0.028 | 0.007 | 0.058 | 0.018 | 0.083 | 0.068 |
| MEDA | 0.971 | 0.007 | 0.059 | 0.017 | 0.029 | 0.007 | 0.064 | 0.020 | 0.094 | 0.077 |
| NEDI | 0.968 | 0.009 | 0.057 | 0.017 | 0.032 | 0.009 | 0.061 | 0.020 | 0.074 | 0.062 |
| NELA | 0.973 | 0.006 | 0.058 | 0.017 | 0.028 | 0.006 | 0.062 | 0.019 | 0.096 | 0.077 |
| NOPE | 0.970 | 0.007 | 0.058 | 0.017 | 0.030 | 0.007 | 0.063 | 0.020 | 0.086 | 0.071 |
| NOSO | 0.972 | 0.007 | 0.059 | 0.017 | 0.028 | 0.007 | 0.063 | 0.019 | 0.097 | 0.079 |
| NPPA | 0.973 | 0.006 | 0.058 | 0.017 | 0.027 | 0.006 | 0.062 | 0.019 | 0.098 | 0.079 |
| PIBA | 0.969 | 0.008 | 0.053 | 0.016 | 0.031 | 0.008 | 0.056 | 0.018 | 0.067 | 0.055 |
| PINR | 0.972 | 0.007 | 0.055 | 0.016 | 0.028 | 0.007 | 0.059 | 0.019 | 0.086 | 0.071 |
| RAGD | 0.970 | 0.007 | 0.067 | 0.019 | 0.030 | 0.007 | 0.072 | 0.022 | 0.114 | 0.092 |
| RAGD_H | 0.968 | 0.008 | 0.058 | 0.017 | 0.032 | 0.008 | 0.062 | 0.019 | 0.080 | 0.064 |
| SACM | 0.968 | 0.008 | 0.059 | 0.017 | 0.032 | 0.008 | 0.063 | 0.019 | 0.087 | 0.070 |
| SEPA | 0.972 | 0.007 | 0.058 | 0.016 | 0.028 | 0.007 | 0.062 | 0.019 | 0.097 | 0.080 |
| SLHL | 0.968 | 0.008 | 0.059 | 0.017 | 0.032 | 0.008 | 0.063 | 0.019 | 0.085 | 0.069 |
| SMCM | 0.968 | 0.007 | 0.061 | 0.018 | 0.032 | 0.007 | 0.065 | 0.020 | 0.092 | 0.073 |
| SNCS | 0.968 | 0.008 | 0.055 | 0.016 | 0.032 | 0.008 | 0.059 | 0.018 | 0.072 | 0.060 |
| SPHL | 0.968 | 0.008 | 0.060 | 0.018 | 0.032 | 0.008 | 0.065 | 0.021 | 0.086 | 0.071 |
| STMI | 0.967 | 0.008 | 0.060 | 0.017 | 0.033 | 0.008 | 0.063 | 0.019 | 0.083 | 0.066 |
| TACM | 0.969 | 0.008 | 0.059 | 0.017 | 0.031 | 0.008 | 0.063 | 0.020 | 0.087 | 0.070 |
| THMB | 0.967 | 0.008 | 0.061 | 0.018 | 0.033 | 0.008 | 0.065 | 0.020 | 0.088 | 0.070 |
| TRCN | 0.968 | 0.008 | 0.057 | 0.017 | 0.032 | 0.008 | 0.061 | 0.019 | 0.077 | 0.063 |
| TRNA | 0.968 | 0.008 | 0.060 | 0.017 | 0.032 | 0.008 | 0.064 | 0.020 | 0.087 | 0.072 |
| VMRD | 0.968 | 0.008 | 0.062 | 0.018 | 0.032 | 0.008 | 0.066 | 0.020 | 0.093 | 0.075 |
| VOFI | 0.970 | 0.007 | 0.063 | 0.018 | 0.030 | 0.007 | 0.068 | 0.021 | 0.103 | 0.082 |
| WIPT | 0.968 | 0.008 | 0.061 | 0.017 | 0.032 | 0.008 | 0.065 | 0.020 | 0.091 | 0.073 |
| YUME | 0.966 | 0.010 | 0.060 | 0.018 | 0.034 | 0.010 | 0.065 | 0.021 | 0.078 | 0.066 |

**Table S2.** Summary statistics for variant sites from the Stacks *Populations* module for 35 Mojave Desert populations of *Chylismia brevipes* genotyped using the *PstI* restriction enzyme.

| **Pop** | **H_o_** | **Var** | **H_exp_** | **Var** | **H_obs_** | **Var** | **π** | **Var** | **F_IS_** | **Var** |
| --- | --- | --- | --- | --- | --- | --- | --- | --- | --- | --- |
| AMRD | 0.967 | 0.008 | 0.054 | 0.016 | 0.033 | 0.008 | 0.058 | 0.018 | 0.068 | 0.055 |
| BMRD | 0.967 | 0.008 | 0.055 | 0.016 | 0.033 | 0.008 | 0.058 | 0.018 | 0.070 | 0.057 |
| BOCA | 0.968 | 0.008 | 0.053 | 0.015 | 0.032 | 0.008 | 0.057 | 0.018 | 0.068 | 0.056 |
| CAGU | 0.965 | 0.013 | 0.051 | 0.017 | 0.035 | 0.013 | 0.059 | 0.023 | 0.049 | 0.048 |
| CIMA | 0.956 | 0.012 | 0.071 | 0.021 | 0.044 | 0.012 | 0.077 | 0.024 | 0.083 | 0.067 |
| DAPA | 0.962 | 0.037 | 0.019 | 0.009 | 0.038 | 0.037 | 0.038 | 0.037 | 0.000 | 0.000 |
| DENS | 0.964 | 0.009 | 0.059 | 0.016 | 0.036 | 0.009 | 0.063 | 0.019 | 0.077 | 0.062 |
| DUGU | 0.966 | 0.008 | 0.057 | 0.016 | 0.034 | 0.008 | 0.061 | 0.018 | 0.077 | 0.062 |
| DVBT | 0.966 | 0.009 | 0.057 | 0.016 | 0.034 | 0.009 | 0.060 | 0.018 | 0.074 | 0.060 |
| EDJR | 0.963 | 0.009 | 0.064 | 0.017 | 0.037 | 0.009 | 0.068 | 0.020 | 0.089 | 0.071 |
| FRAN | 0.971 | 0.007 | 0.052 | 0.015 | 0.029 | 0.007 | 0.056 | 0.017 | 0.073 | 0.058 |
| GAWA | 0.970 | 0.008 | 0.053 | 0.016 | 0.031 | 0.008 | 0.057 | 0.018 | 0.073 | 0.058 |
| HITW | 0.967 | 0.008 | 0.057 | 0.016 | 0.033 | 0.008 | 0.061 | 0.018 | 0.079 | 0.064 |
| JUWA | 0.966 | 0.011 | 0.054 | 0.017 | 0.034 | 0.011 | 0.062 | 0.023 | 0.059 | 0.054 |
| LAME | 0.967 | 0.008 | 0.058 | 0.017 | 0.033 | 0.008 | 0.062 | 0.019 | 0.081 | 0.065 |
| LAVA | 0.966 | 0.008 | 0.058 | 0.017 | 0.034 | 0.008 | 0.062 | 0.019 | 0.078 | 0.063 |
| NEDI | 0.971 | 0.008 | 0.051 | 0.016 | 0.029 | 0.008 | 0.055 | 0.018 | 0.066 | 0.055 |
| NOSO | 0.960 | 0.010 | 0.062 | 0.017 | 0.040 | 0.010 | 0.065 | 0.019 | 0.074 | 0.061 |
| PIBA | 0.976 | 0.006 | 0.045 | 0.014 | 0.024 | 0.006 | 0.048 | 0.016 | 0.065 | 0.052 |
| PINR | 0.964 | 0.009 | 0.059 | 0.017 | 0.036 | 0.009 | 0.063 | 0.019 | 0.076 | 0.061 |
| RAGD | 0.960 | 0.009 | 0.072 | 0.019 | 0.040 | 0.009 | 0.076 | 0.022 | 0.103 | 0.081 |
| RAGD_H | 0.974 | 0.006 | 0.057 | 0.017 | 0.026 | 0.006 | 0.061 | 0.019 | 0.093 | 0.074 |
| SACM | 0.973 | 0.006 | 0.054 | 0.016 | 0.027 | 0.006 | 0.058 | 0.018 | 0.088 | 0.070 |
| SEPA | 0.963 | 0.009 | 0.063 | 0.017 | 0.037 | 0.009 | 0.067 | 0.019 | 0.088 | 0.071 |
| SLHL | 0.976 | 0.006 | 0.051 | 0.015 | 0.024 | 0.006 | 0.055 | 0.017 | 0.085 | 0.069 |
| SNCS | 0.973 | 0.006 | 0.051 | 0.015 | 0.027 | 0.006 | 0.054 | 0.017 | 0.076 | 0.061 |
| SPHL | 0.969 | 0.008 | 0.058 | 0.017 | 0.031 | 0.008 | 0.062 | 0.020 | 0.081 | 0.067 |
| STMI | 0.971 | 0.006 | 0.059 | 0.017 | 0.029 | 0.006 | 0.063 | 0.019 | 0.095 | 0.075 |
| TACM | 0.970 | 0.007 | 0.056 | 0.016 | 0.030 | 0.007 | 0.060 | 0.018 | 0.084 | 0.066 |
| THMB | 0.967 | 0.008 | 0.060 | 0.017 | 0.033 | 0.008 | 0.064 | 0.019 | 0.087 | 0.068 |
| TRCN | 0.968 | 0.008 | 0.055 | 0.016 | 0.032 | 0.008 | 0.059 | 0.018 | 0.074 | 0.060 |
| TRNA | 0.965 | 0.008 | 0.060 | 0.017 | 0.035 | 0.008 | 0.064 | 0.019 | 0.084 | 0.067 |
| VMRD | 0.967 | 0.008 | 0.060 | 0.017 | 0.033 | 0.008 | 0.064 | 0.019 | 0.089 | 0.070 |
| WIPT | 0.967 | 0.008 | 0.059 | 0.017 | 0.033 | 0.008 | 0.063 | 0.019 | 0.084 | 0.067 |
| YUME | 0.971 | 0.008 | 0.054 | 0.016 | 0.029 | 0.008 | 0.058 | 0.019 | 0.074 | 0.061 |

**Appendix S3.**

**Figure 1.** Discriminant analysis of principal components (DAPC) conducted on the *SbfI* dataset. **A.** Mapped colors indicate the posterior population probabilities aggregated across individuals at a sample location (i.e., different colors represent different ancestral populations). **B**. BIC criterion for choosing number of clusters. **C.** Scatter plots from the DAPC analysis.

**A)**


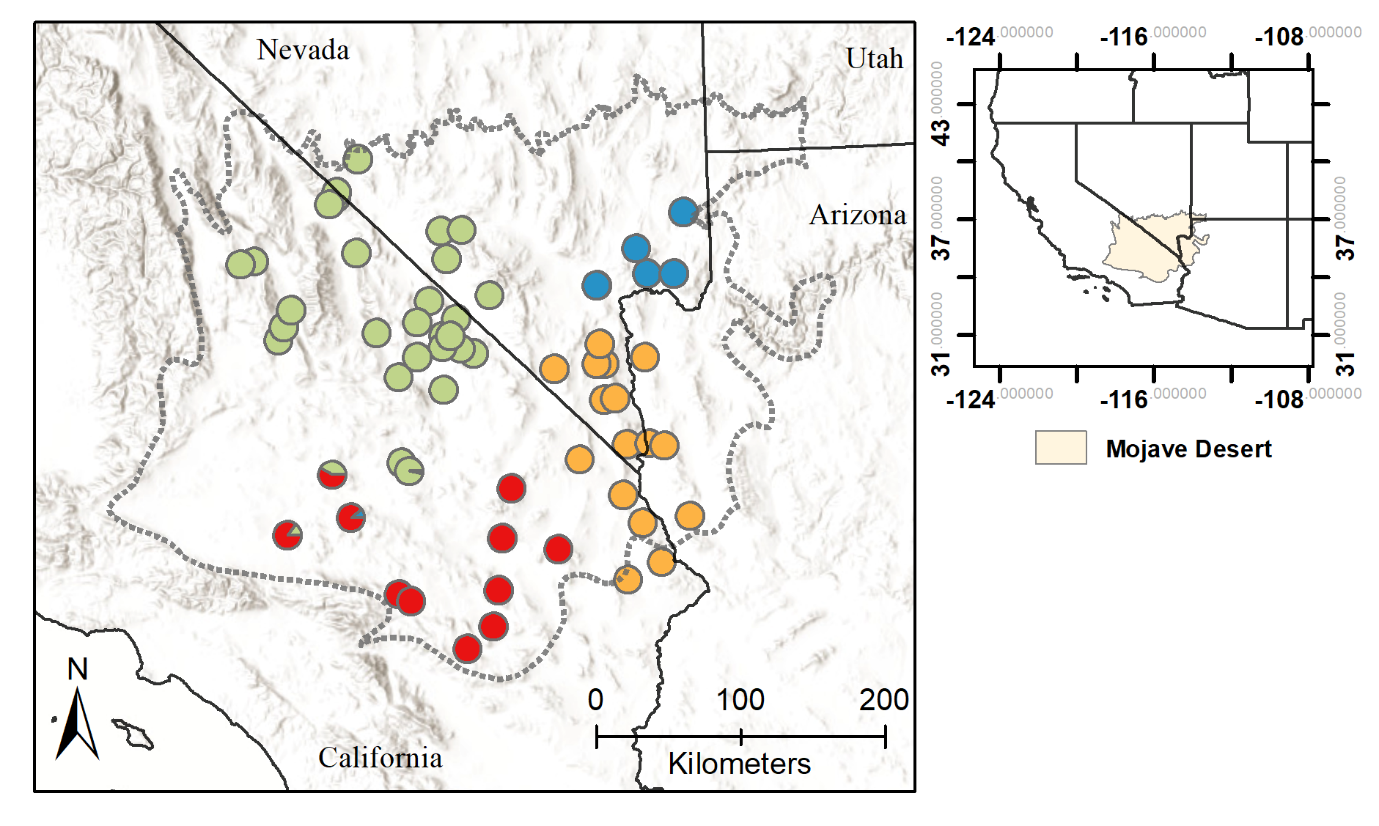


**B)**


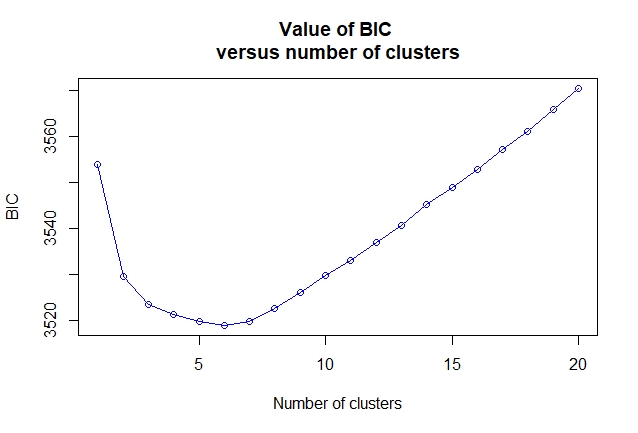


**C)**

**
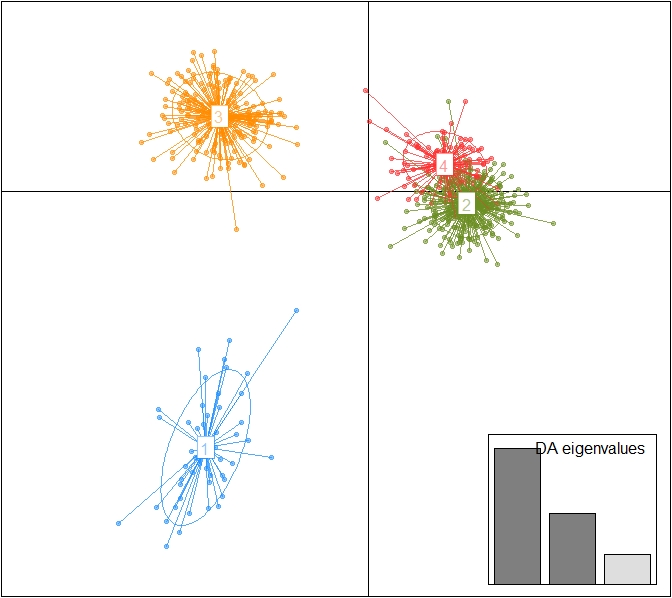
**

**
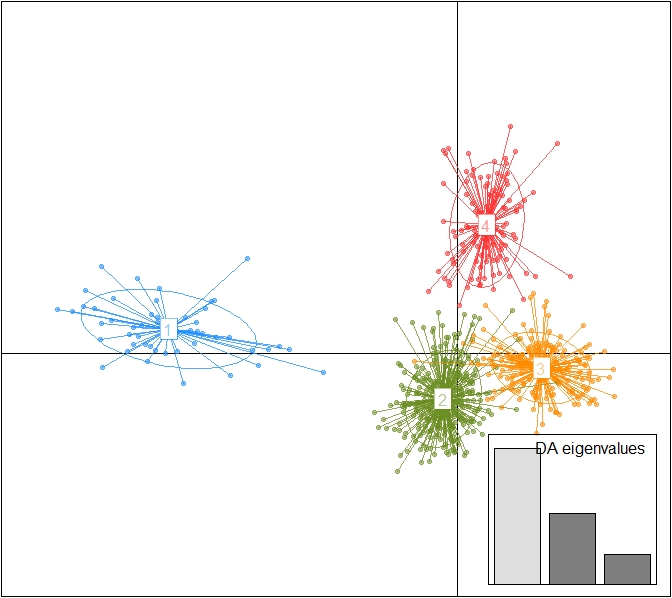
**

**Figure 2.** Discriminant analysis of principal components (DAPC) conducted on the *PstI* dataset. Mapped colors indicate the posterior population probabilities aggregated across individuals at a sample location (i.e., different colors represent different ancestral populations).

**A)**


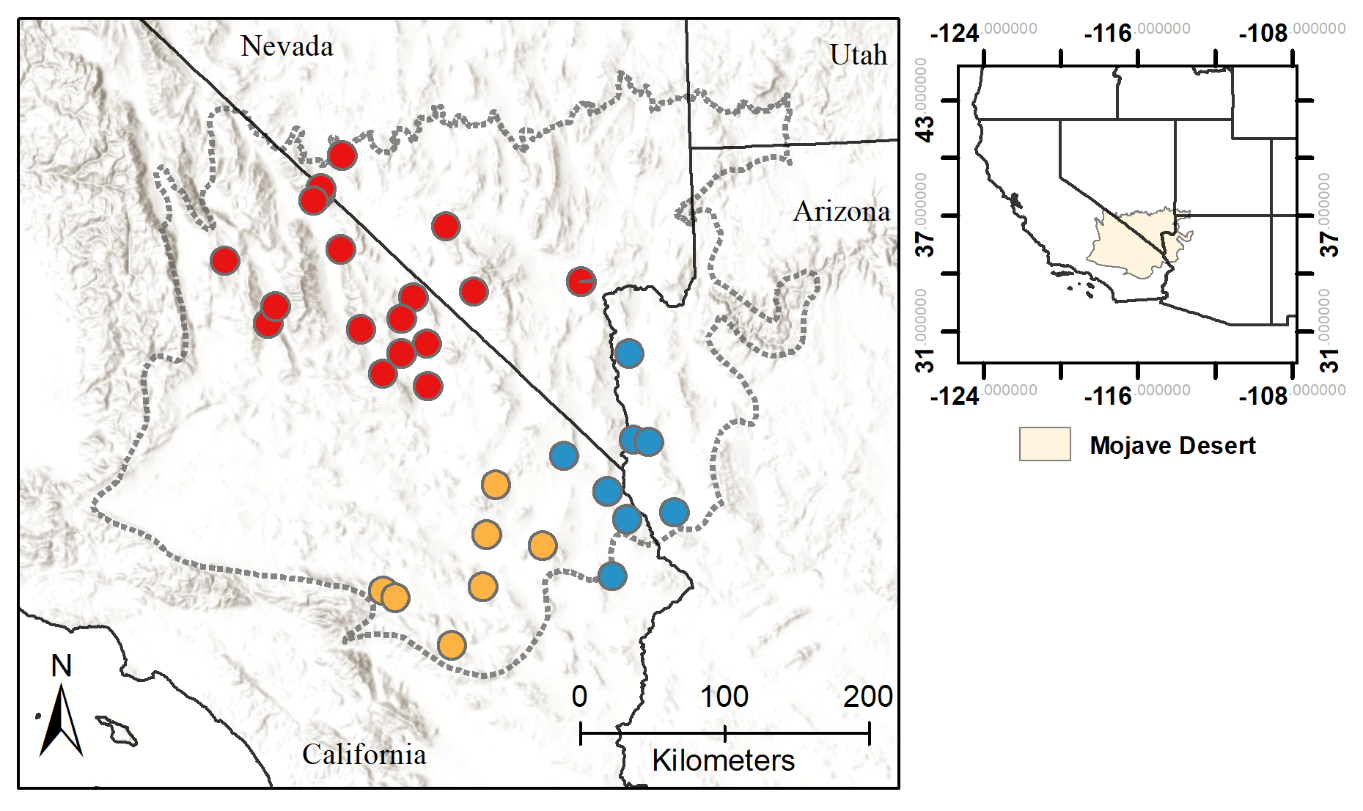


**B)**


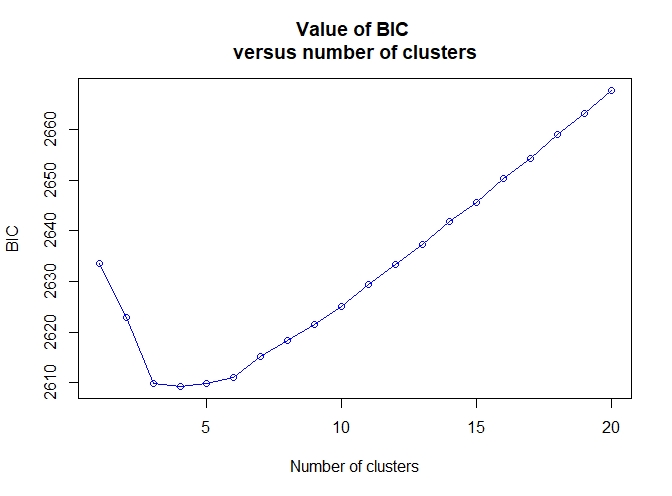


**C)**

**
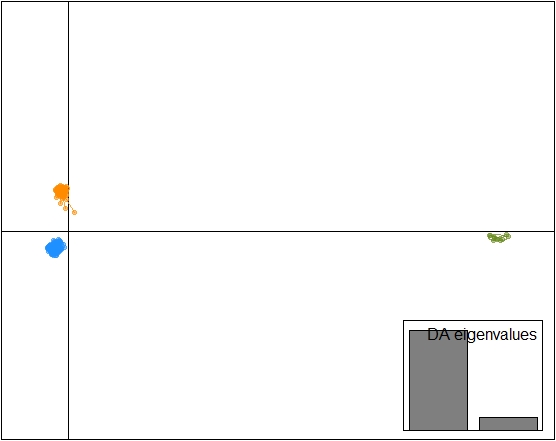
**

**Appendix S4.**

Table 1. Potentially adaptive loci based on RDA and BayeScEnv for the SbfI dataset. The ‘predictor’ and ‘correlation’ columns indicate the variable to which each candidate SNP is maximally correlated. The last four columns provide method-specific results.

|  |  |  |  |  | **RDA** |  | **BayeScEnv** | |
| --- | --- | --- | --- | --- | --- | --- | --- | --- |
| **SNP** | **RDA** | **Bayescenv** | **predictor** | **correlation** | **Loading** | **Axis #** | **Fst** | **Qval** |
| 3325_79 | 1 | 1 | BD | 0.418 | -0.34955 | 2 | 0.30489 | 0.001 |
| 143773_54 | 0 | 1 | dtrange | 0.527 | - | - | 0.47181 | 0.010052 |
| 3050_51 | 0 | 1 | dtrange | 0.449 | - | - | 0.71995 | 0.0019 |
| 4161_52 | 0 | 1 | dtrange | 0.301 | - | - | 0.31928 | 0.053731 |
| 184207_65 | 0 | 1 | Fall.PPT | 0.512 | - | - | 0.37249 | 0.017163 |
| 273295_53 | 0 | 1 | Fall.PPT | 0.467 | - | - | 0.5107 | 0.003467 |
| 279798_23 | 0 | 1 | Fall.PPT | 0.509 | - | - | 0.37221 | 0.004801 |
| 3441_8 | 0 | 1 | Fall.PPT | 0.452 | - | - | 0.34598 | 0.022071 |
| 3795_41 | 0 | 1 | Fall.PPT | 0.473 | - | - | 0.24844 | 0.046109 |
| 4023_46 | 0 | 1 | Fall.PPT | 0.507 | - | - | 0.40837 | 0.041694 |
| 7699_38 | 0 | 1 | Fall.PPT | 0.404 | - | - | 0.3351 | 0.006401 |
| 107560_78 | 1 | 0 | Fall.PPT | 0.534 | -0.64867 | 1 | - | - |
| 130302_53 | 1 | 0 | Fall.PPT | 0.392 | 0.284917 | 3 | - | - |
| 198759_55 | 1 | 0 | Fall.PPT | 0.616 | -0.65363 | 1 | - | - |
| 204199_84 | 1 | 0 | Fall.PPT | 0.668 | 0.631417 | 1 | - | - |
| 628_58 | 1 | 0 | Fall.PPT | 0.653 | 0.610327 | 1 | - | - |
| 7003_50 | 1 | 0 | Fall.PPT | 0.586 | -0.67347 | 1 | - | - |
| 7959_42 | 1 | 0 | Fall.PPT | 0.636 | -0.56105 | 1 | - | - |
| 294996_75 | 0 | 1 | pcv | 0.315 | - | - | 0.3147 | 0.031606 |
| 299326_40 | 0 | 1 | pcv | 0.28 | - | - | 0.76529 | 0.015203 |
| 143782_32 | 1 | 0 | pcv | 0.365 | 0.367136 | 2 | - | - |
| 1291_41 | 1 | 0 | Sand | 0.488 | -0.23715 | 3 | - | - |
| 2901_10 | 1 | 0 | Sand | 0.367 | -0.23816 | 3 | - | - |
| 6092_21 | 0 | 1 | TPI | 0.26 | - | - | 0.5588 | 0.013536 |
| 2239_74 | 0 | 1 | wmt | 0.52 | - | - | 0.33223 | 0.029656 |
| 276374_50 | 0 | 1 | wmt | 0.548 | - | - | 0.30928 | 0.011002 |
| 5264_38 | 0 | 1 | wmt | 0.59 | - | - | 0.34305 | 0.039408 |
| 142455_51 | 1 | 0 | wmt | 0.558 | 0.364858 | 2 | - | - |
| 162974_48 | 1 | 0 | wmt | 0.551 | -0.3384 | 2 | - | - |
| 195591_60 | 1 | 0 | wmt | 0.463 | -0.4124 | 2 | - | - |
| 201835_61 | 1 | 0 | wmt | 0.604 | 0.438316 | 2 | - | - |
| 144342_14 | 0 | 1 | wpratio | 0.534 | - | - | 0.3645 | 0.006501 |
| 196_22 | 0 | 1 | wpratio | 0.535 | - | - | 0.34803 | 0.004401 |
| 2060_5 | 1 | 1 | wpratio | 0.587 | -0.35855 | 2 | 0.29521 | 0.032957 |
| 104825_84 | 1 | 0 | wpratio | 0.515 | 0.39332 | 2 | - | - |
| 172802_81 | 1 | 0 | wpratio | 0.433 | -0.24888 | 3 | - | - |
| 183106_52 | 1 | 0 | wpratio | 0.287 | 0.34404 | 2 | - | - |
| 2443_62 | 1 | 0 | wpratio | 0.383 | -0.4227 | 2 | - | - |
| 249225_30 | 1 | 0 | wpratio | 0.433 | -0.35067 | 2 | - | - |
| 2629_65 | 1 | 0 | wpratio | 0.575 | -0.36706 | 2 | - | - |
| 274181_70 | 1 | 0 | wpratio | 0.451 | 0.3892 | 2 | - | - |
| 4176_18 | 1 | 0 | wpratio | 0.373 | -0.24837 | 3 | - | - |
| 5933_4 | 1 | 0 | wpratio | 0.385 | 0.267437 | 3 | - | - |
| 659_67 | 1 | 0 | wpratio | 0.276 | -0.3948 | 2 | - | - |

**Table 2**. Potentially adaptive loci based on RDA and BayeScEnv for the PstI dataset. The ‘predictor’ and ‘correlation’ columns indicate the variable to which each candidate SNP is maximally correlated. The last four columns provide method-specific results.

|  |  |  |  |  | **RDA** |  | **BayeScEnv** | |
| --- | --- | --- | --- | --- | --- | --- | --- | --- |
| **SNP** | **RDA** | **BayescEnv** | **predictor** | **correlation** | **loading** | **axis #** | **qval** | **Fst** |
| 6157_76 | 0 | 1 | AMP | 0.375 | - | - | 0.007302 | 0.37986 |
| 22502_78 | 1 | 0 | AMP | 0.376 | -0.26815 | 2 | - | - |
| 6971_46 | 0 | 1 | BD | 0.656 | - | - | 0.056564 | 0.48905 |
| 7844_27 | 0 | 1 | BD | 0.426 | - | - | 0.008002 | 0.749 |
| 3305_75 | 1 | 0 | BD | 0.377 | 0.197981 | 3 | - | - |
| 5001_49 | 0 | 1 | DTrange | 0.426 | - | - | 0.043809 | 0.28027 |
| 10593_70 | 0 | 1 | Fall.PPT | 0.52 | - | - | 0.025005 | 0.40357 |
| 13184_8 | 0 | 1 | Fall.PPT | 0.464 | - | - | 0.05045 | 0.26184 |
| 1706_35 | 0 | 1 | Fall.PPT | 0.342 | - | - | 0.020844 | 0.34269 |
| 17995_41 | 1 | 1 | Fall.PPT | 0.675 | 0.436627 | 1 | 0.002 | 0.46665 |
| 20462_45 | 1 | 1 | Fall.PPT | 0.581 | 0.437968 | 1 | 0.007101 | 0.36666 |
| 21582_89 | 0 | 1 | Fall.PPT | 0.535 | - | - | 0.040558 | 0.3629 |
| 2188_81 | 0 | 1 | Fall.PPT | 0.531 | - | - | 0.004601 | 0.34791 |
| 2252_90 | 1 | 1 | Fall.PPT | 0.547 | 0.436894 | 1 | 0.0002 | 0.7986 |
| 3263_70 | 0 | 1 | Fall.PPT | 0.503 | - | - | 0.031424 | 0.37636 |
| 423923_84 | 0 | 1 | Fall.PPT | 0.532 | - | - | 0.004534 | 0.44101 |
| 4312_81 | 0 | 1 | Fall.PPT | 0.521 | - | - | 0.026272 | 0.33814 |
| 4629_34 | 0 | 1 | Fall.PPT | 0.514 | - | - | 0.004801 | 0.30154 |
| 523774_59 | 1 | 1 | Fall.PPT | 0.61 | -0.38557 | 1 | 0.004801 | 0.31136 |
| 546531_10 | 0 | 1 | Fall.PPT | 0.604 | - | - | 0.002801 | 0.54646 |
| 582886_88 | 0 | 1 | Fall.PPT | 0.554 | - | - | 0.018354 | 0.33517 |
| 5868_8 | 1 | 1 | Fall.PPT | 0.615 | 0.421927 | 1 | 0.019115 | 0.2768 |
| 6094_11 | 0 | 1 | Fall.PPT | 0.575 | - | - | 0.000333 | 0.37155 |
| 6408_37 | 0 | 1 | Fall.PPT | 0.682 | - | - | 0.022004 | 0.33806 |
| 6798_82 | 0 | 1 | Fall.PPT | 0.568 | - | - | 0.037636 | 0.31328 |
| 8369_41 | 0 | 1 | Fall.PPT | 0.62 | - | - | 0.025605 | 0.43821 |
| 8892_8 | 1 | 1 | Fall.PPT | 0.616 | 0.418816 | 1 | 0.040708 | 0.44392 |
| 1601_38 | 1 | 0 | Fall.PPT | 0.55 | 0.402141 | 1 | - | - |
| 9519_15 | 1 | 0 | Fall.PPT | 0.628 | 0.419534 | 1 | - | - |
| 1743_13 | 0 | 1 | PCV | 0.588 | - | - | 0.005601 | 0.30613 |
| 4424_59 | 0 | 1 | PCV | 0.48 | - | - | 0.033335 | 0.27023 |
| 4317_78 | 1 | 0 | PCV | 0.385 | -0.28431 | 2 | - | - |
| 2493_48 | 0 | 1 | Sand | 0.479 | - | - | 0.024187 | 0.2946 |
| 5045_71 | 0 | 1 | Sand | 0.66 | - | - | 0.023455 | 0.23661 |
| 8134_36 | 0 | 1 | Sand | 0.388 | - | - | 0.030175 | 0.37168 |
| 2473_75 | 1 | 0 | Sand | 0.688 | 0.204405 | 3 | - | - |
| 29814_25 | 1 | 0 | Sand | 0.467 | 0.181119 | 3 | - | - |
| 4109_82 | 1 | 0 | Sand | 0.56 | 0.218087 | 3 | - | - |
| 4301_38 | 1 | 0 | Sand | 0.456 | 0.182761 | 3 | - | - |
| 55249_44 | 1 | 0 | Sand | 0.474 | 0.232203 | 3 | - | - |
| 6490_86 | 1 | 0 | Sand | 0.323 | -0.27643 | 2 | - | - |
| 13478_82 | 0 | 1 | SMT | 0.696 | - | - | 0.0001 | 0.5428 |
| 21816_8 | 0 | 1 | SMT | 0.346 | - | - | 0.031006 | 0.28678 |
| 4527_43 | 0 | 1 | SMT | 0.67 | - | - | 0.027272 | 0.46281 |
| 4621_17 | 0 | 1 | SMT | 0.425 | - | - | 0.011642 | 0.30169 |
| 7155_41 | 0 | 1 | SMT | 0.463 | - | - | 0.020337 | 0.25295 |
| 14347_59 | 0 | 1 | WMT | 0.647 | - | - | 0.002301 | 0.26595 |
| 1516_71 | 1 | 1 | WMT | 0.631 | 0.426665 | 1 | 0.032757 | 0.29879 |
| 16239_41 | 0 | 1 | WMT | 0.472 | - | - | 0.012736 | 0.32639 |
| 1695_40 | 0 | 1 | WMT | 0.615 | - | - | 0.008602 | 0.25888 |
| 20315_31 | 0 | 1 | WMT | 0.574 | - | - | 0.028383 | 0.35535 |
| 20965_41 | 0 | 1 | WMT | 0.446 | - | - | 0.044484 | 0.31493 |
| 4262_69 | 0 | 1 | WMT | 0.583 | - | - | 0.041728 | 0.29045 |
| 53265_25 | 0 | 1 | WMT | 0.575 | - | - | 0.009802 | 0.69585 |
| 5656_78 | 0 | 1 | WMT | 0.581 | - | - | 0.044142 | 0.39203 |
| 57416_74 | 0 | 1 | WMT | 0.493 | - | - | 0.036341 | 0.46051 |
| 7425_41 | 0 | 1 | WMT | 0.558 | - | - | 0.018404 | 0.32021 |
| 7331_50 | 1 | 0 | WMT | 0.515 | 0.407862 | 1 | - | - |
| 10937_31 | 1 | 1 | WPratio | 0.741 | -0.35224 | 2 | 0.003801 | 0.34604 |
| 12365_30 | 0 | 1 | WPratio | 0.598 | - | - | 0.002 | 0.3934 |
| 1505_71 | 0 | 1 | WPratio | 0.726 | - | - | 0.043126 | 0.36159 |
| 1910_44 | 0 | 1 | WPratio | 0.501 | - | - | 0.005668 | 0.35341 |
| 19636_85 | 0 | 1 | WPratio | 0.57 | - | - | 0.012669 | 0.43114 |
| 21083_90 | 1 | 1 | WPratio | 0.572 | 0.298287 | 2 | 0.041308 | 0.40743 |
| 21550_73 | 0 | 1 | WPratio | 0.505 | - | - | 0.013703 | 0.31632 |
| 2193_81 | 0 | 1 | WPratio | 0.632 | - | - | 0.021164 | 0.34781 |
| 22113_51 | 0 | 1 | WPratio | 0.656 | - | - | 0.017629 | 0.30696 |
| 22526_37 | 0 | 1 | WPratio | 0.617 | - | - | 0.015746 | 0.26979 |
| 3268_73 | 1 | 1 | WPratio | 0.37 | -0.28429 | 2 | 0.030373 | 0.36851 |
| 3320_77 | 0 | 1 | WPratio | 0.529 | - | - | 0.014203 | 0.49999 |
| 4349_29 | 1 | 1 | WPratio | 0.72 | -0.2795 | 2 | 0.028606 | 0.27372 |
| 5828_29 | 0 | 1 | WPratio | 0.547 | - | - | 0.004401 | 0.30827 |
| 583562_37 | 0 | 1 | WPratio | 0.589 | - | - | 0.03254 | 0.26994 |
| 12214_75 | 1 | 0 | WPratio | 0.514 | -0.29989 | 2 | - | - |
| 18115_71 | 1 | 0 | WPratio | 0.634 | -0.33961 | 2 | - | - |
| 20908_29 | 1 | 0 | WPratio | 0.38 | -0.26415 | 2 | - | - |
| 24784_75 | 1 | 0 | WPratio | 0.655 | -0.30395 | 2 | - | - |
| 3369_12 | 1 | 0 | WPratio | 0.408 | -0.29028 | 2 | - | - |
| 3941_39 | 1 | 0 | WPratio | 0.736 | -0.30992 | 2 | - | - |
| 4725_44 | 1 | 0 | WPratio | 0.453 | -0.29171 | 2 | - | - |
| 49701_70 | 1 | 0 | WPratio | 0.695 | -0.28021 | 2 | - | - |
| 536_77 | 1 | 0 | WPratio | 0.524 | -0.29209 | 2 | - | - |
| 5361_82 | 1 | 0 | WPratio | 0.725 | -0.28814 | 2 | - | - |
| 6909_47 | 1 | 0 | WPratio | 0.452 | -0.32233 | 2 | - | - |
| 7307_75 | 1 | 0 | WPratio | 0.489 | -0.26169 | 2 | - | - |
| 99073_69 | 1 | 0 | WPratio | 0.553 | -0.29665 | 2 | - | - |

**Appendix S5. Figure 1****.** Comparison of GDM and GF transformed predictors for the *SbfI* dataset. GDM and GF transformations put each variable on a scale of genetic importance. Layers are displayed as stretched in ArcGIS Pro using 2.5 standard deviations to show differences in overall spatial pattern. Colors represent gradients in allele frequencies associated with each environmental variable, and areas with similar colors are likely to have similar genetic composition.


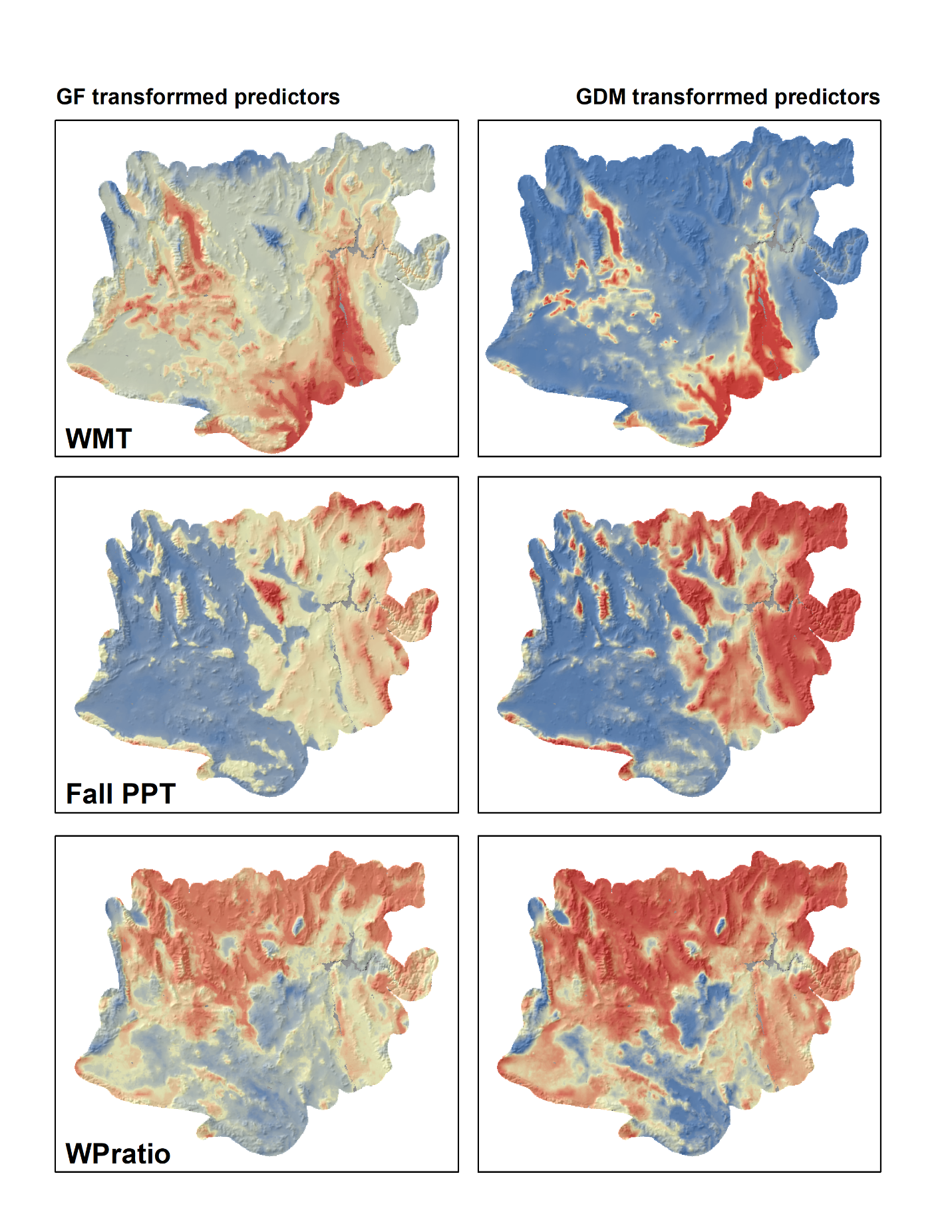


**Appendix S6.**

**Figure 1**. Response curves from gradient forest model of environmental associations for the *PstI* dataset. Panels display the six terms with highest relative importance in the model.


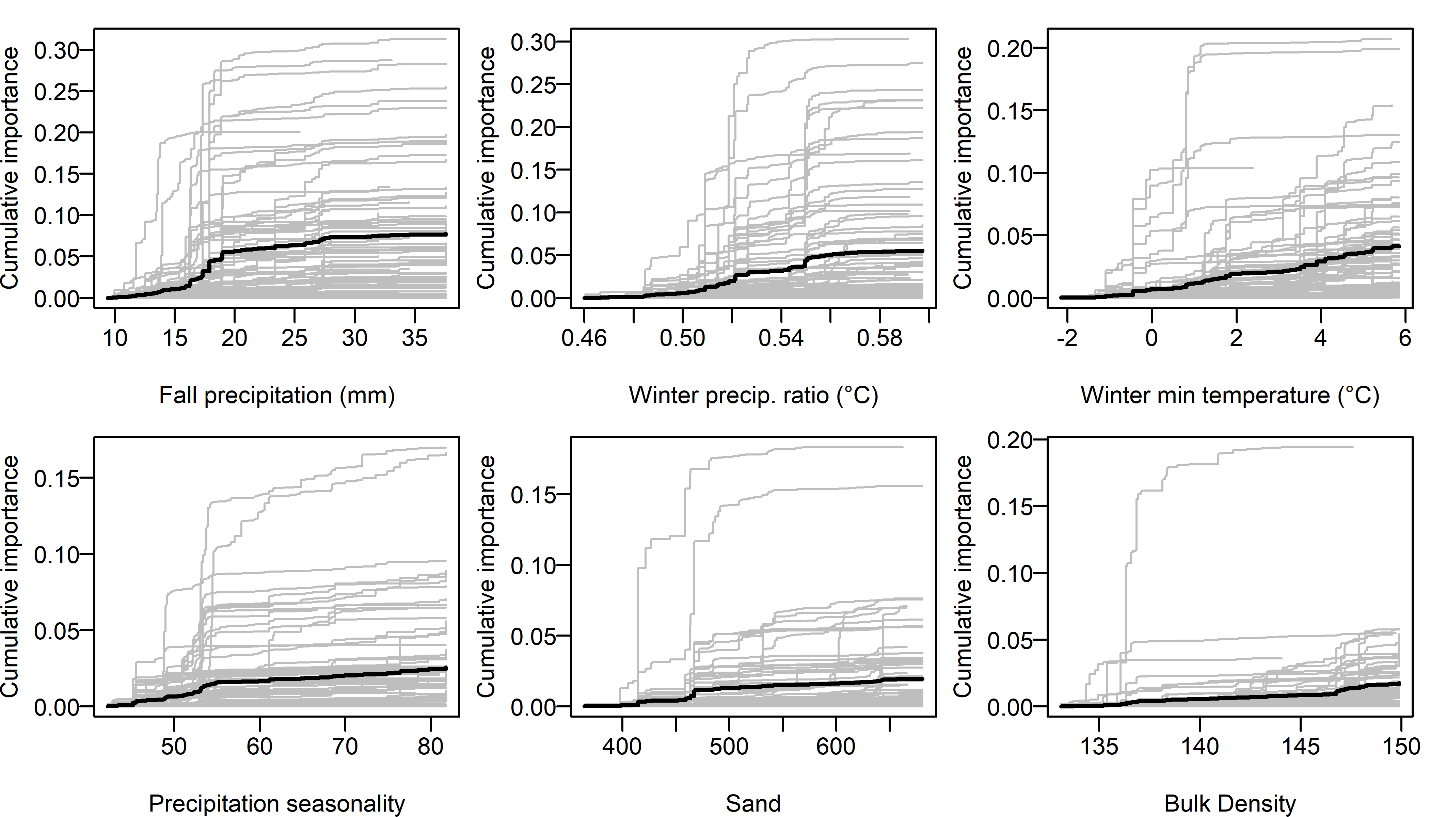


**Figure 2**. Response curves from GDM model of environmental associations for the *PstI* dataset. Dashed lines indicate standard deviations derived from bootstrapping the GDM models with 999 permutations. Panels display the six terms with highest relative importance.


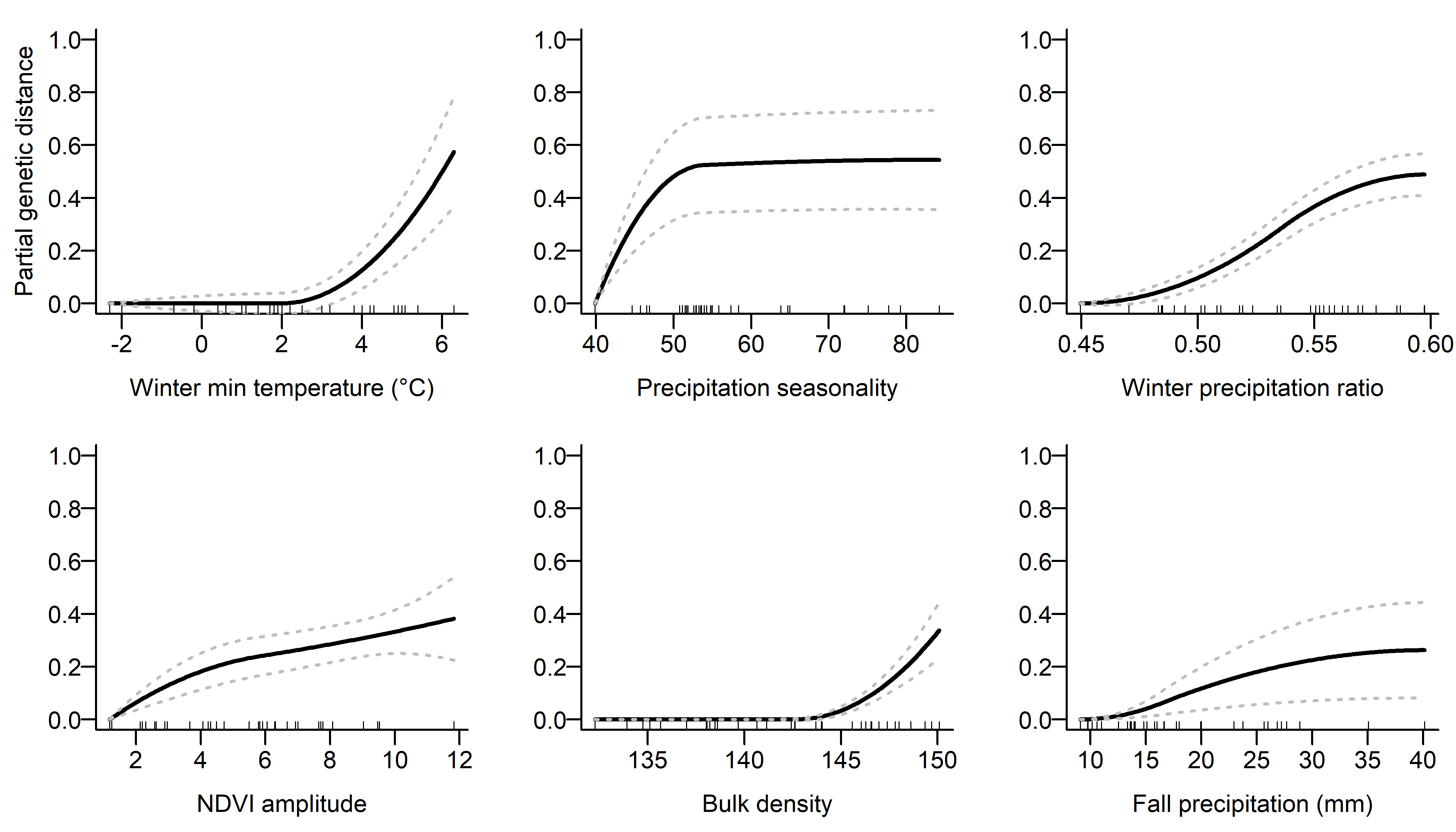


**Appendix S7.**

**Figure 1**. Response curves from the best-fit GDM model incorporating habitat resistance (as a population pairwise distance matrix based on least cost paths) for the *SbfI* dataset. This model explained 56% of the deviance in potentially adaptive allele frequencies. Panels display the six terms with highest relative importance.


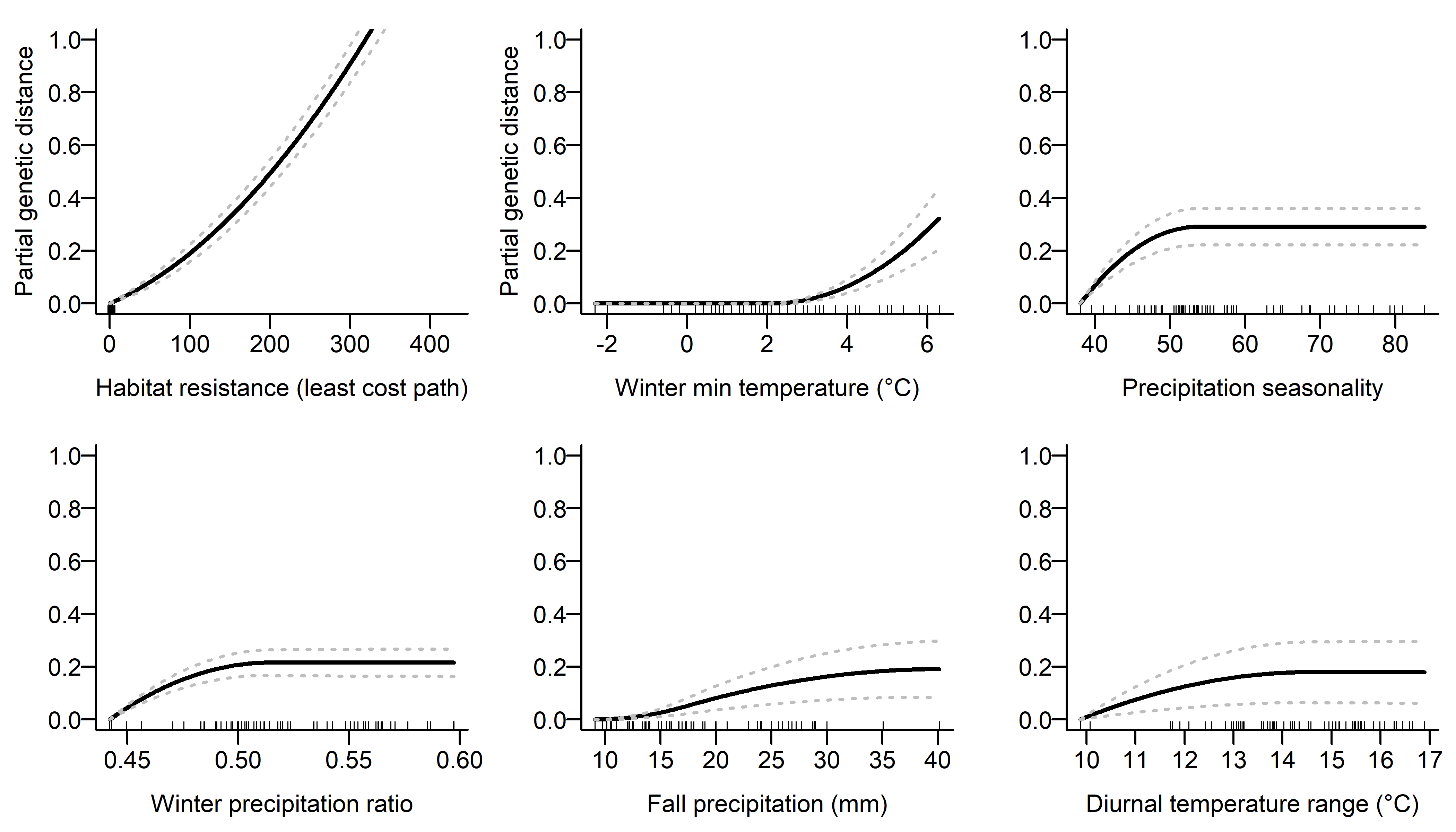


**Appendix S8.**

**Figure 1**. Seed transfer zones for *Chylismia brevipes* in the Mojave Desert. Seed transfer zones aim to identify geographic areas within which seeds may be transferred with limited risk of maladaptation. Four seed transfer zones are provided in this map. Each zone is identified by a unique color.


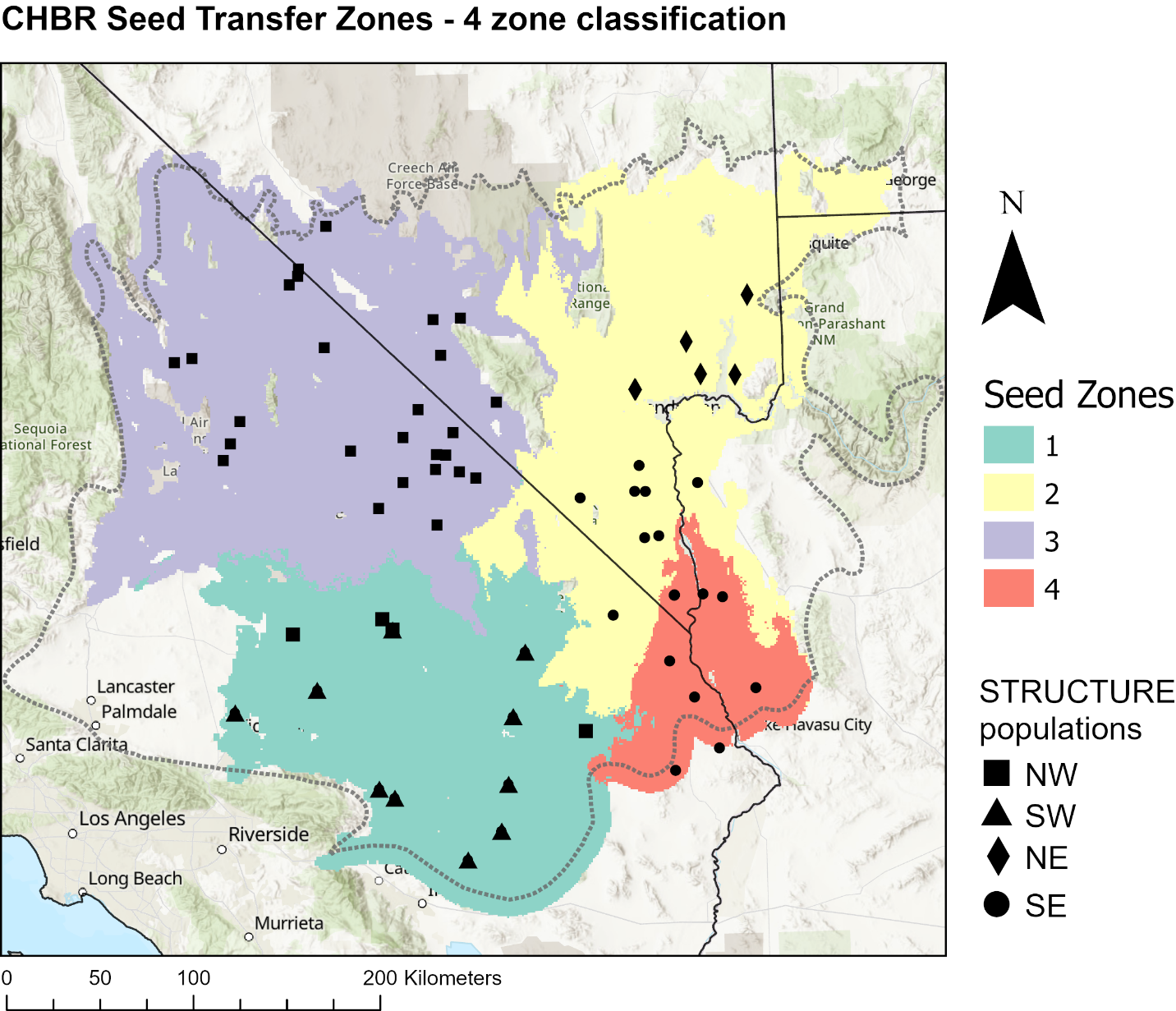


**Figure 2**. Seed transfer zones for *Chylismia brevipes* in the Mojave Desert. Seed transfer zones aim to identify geographic areas within which seeds may be transferred with limited risk of maladaptation. Six seed transfer zones are provided in this map. Each zone is identified by a unique color.


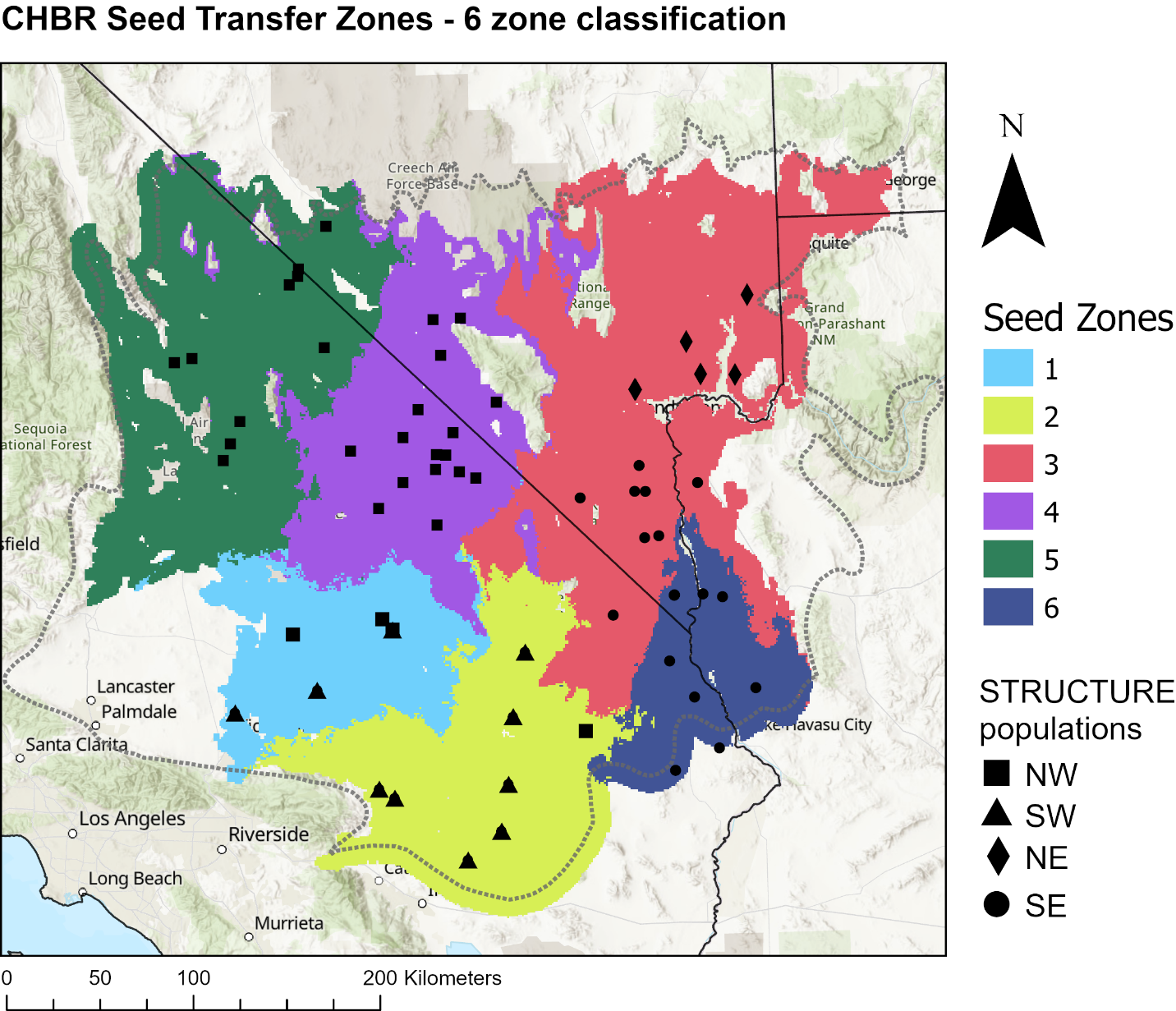


**Appendix S9. Figure 1**. Genomic offset calculated from GDM models for the *SbfI* and *PstI* datasets. The offset is a measure of susceptibility to climate change, indicating where rapid changes in allele frequencies would be necessary to track changes in climate based on the modelled environmental associations.


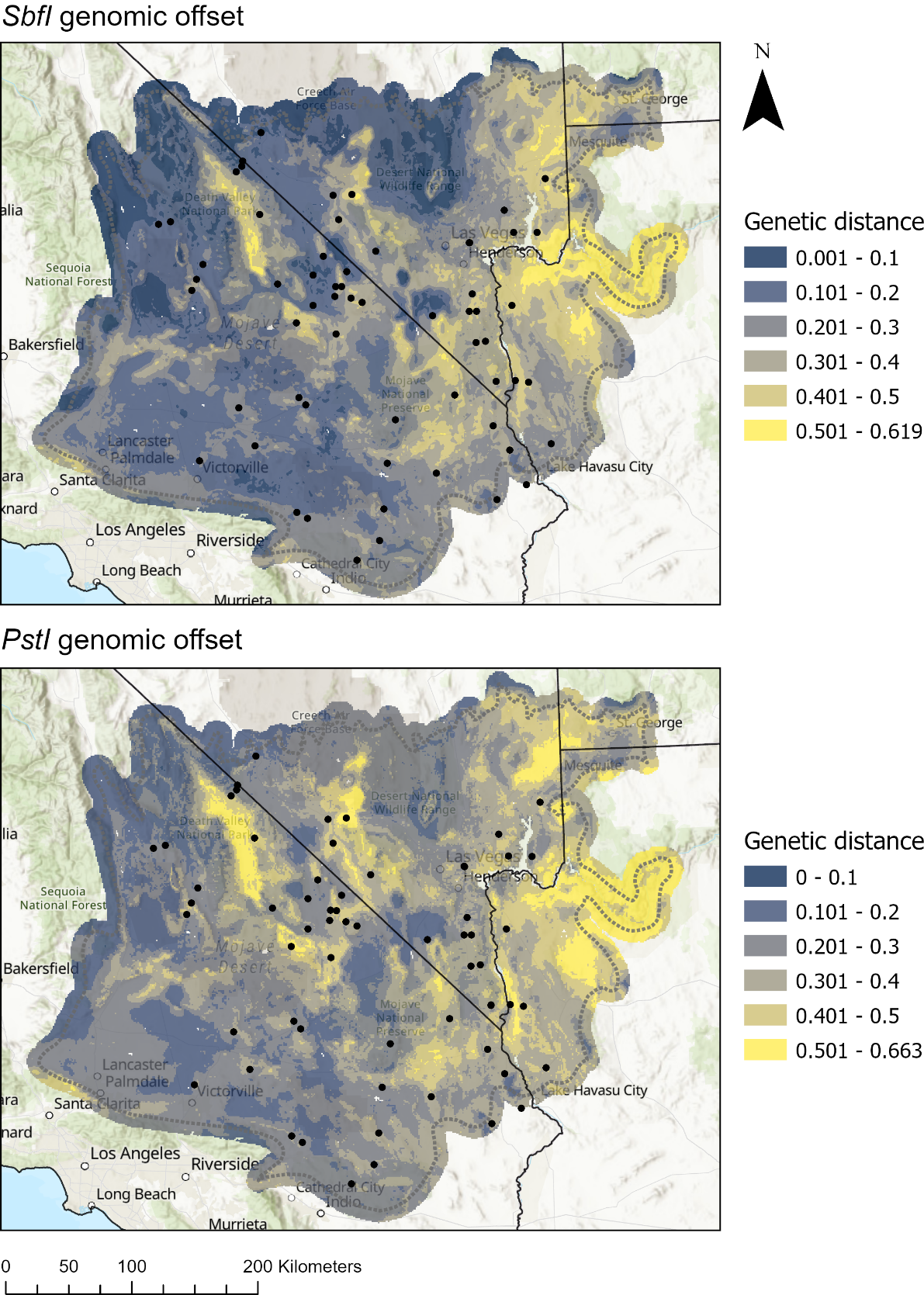

Supplement: Supplementary file 1 — Data S1. [file EVA-17-e70046-s001.docx]
